# Network-based methods for psychometric data of eating disorders: A systematic review

**DOI:** 10.1371/journal.pone.0276341

**Published:** 2022-10-31

**Authors:** Clara Punzi, Manuela Petti, Paolo Tieri

**Affiliations:** 1 Data Science MSc Program, Sapienza University of Rome, Rome, Italy; 2 DIAG Department of Computer, Control and Management Engineering, Sapienza University of Rome, Rome, Italy; 3 CNR National Research Council, IAC Institute for Applied Computing, Rome, Italy; University of Padova, ITALY

## Abstract

**Background:**

Network science represents a powerful and increasingly promising method for studying complex real-world problems. In the last decade, it has been applied to psychometric data in the attempt to explain psychopathologies as complex systems of causally interconnected symptoms. One category of mental disorders, relevant for their severity, incidence and multifaceted structure, is that of eating disorders (EDs), serious disturbances that negatively affect a person’s eating behavior.

**Aims:**

We aimed to review the corpus of psychometric network analysis methods by scrutinizing a large sample of network-based studies that exploit psychometric data related to EDs. A particular focus is given to the description of the methodologies for network estimation, network description and network stability analysis providing also a review of the statistical software packages currently used to carry out each phase of the network estimation and analysis workflow. Moreover, we try to highlight aspects with potential clinical impact such as core symptoms, influences of external factors, comorbidities, and related changes in network structure and connectivity across both time and subpopulations.

**Methods:**

A systematic search was conducted (February 2022) on three different literature databases to identify 57 relevant research articles. The exclusion criteria comprehended studies not based on psychometric data, studies not using network analysis, studies with different aims or not focused on ED, and review articles.

**Results:**

Almost all the selected 57 papers employed the same analytical procedures implemented in a collection of *R* packages specifically designed for psychometric network analysis and are mostly based on cross-sectional data retrieved from structured psychometric questionnaires, with just few exemptions of panel data. Most of them used the same techniques for all phases of their analysis. In particular, a pervasive use of the Gaussian Graphical Model with LASSO regularization was registered for in network estimation step. Among the clinically relevant results, we can include the fact that all papers found strong symptom interconnections between specific and nonspecific ED symptoms, suggesting that both types should therefore be addressed by clinical treatment.

**Conclusions:**

We here presented the largest and most comprehensive review to date about psychometric network analysis methods. Although these methods still need solid validation in the clinical setting, they have already been able to show many strengths and important results, as well as great potentials and perspectives, which have been analyzed here to provide suggestions on their use and their possible improvement.

## Introduction

**Fig 1:**
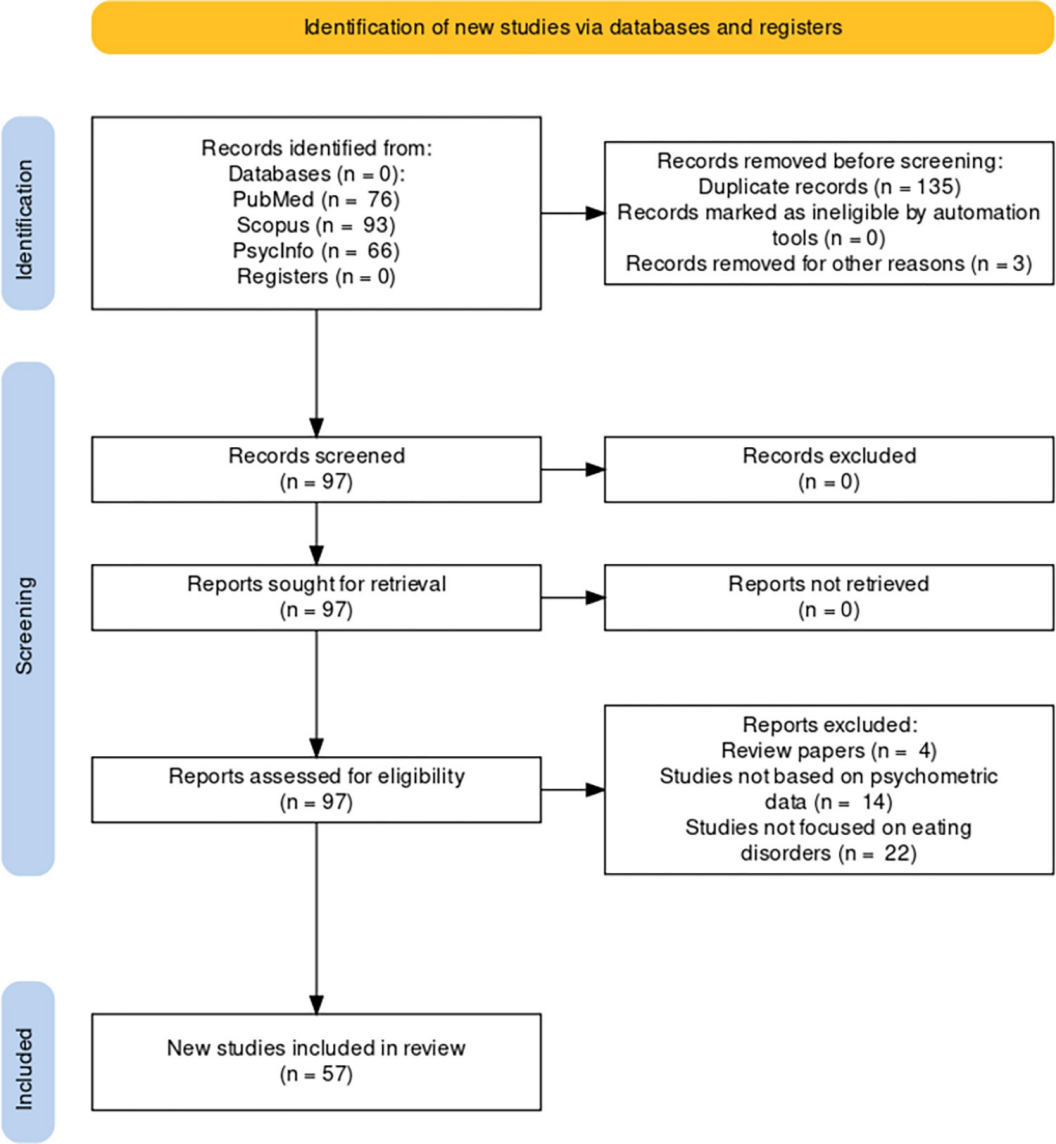
PRISMA 2020 flow diagram for systematic review. https://doi.org/10.1371/journal.pone.0267285.g005.

In the present work, we aim to describe the current state-of-the-art of the network conceptualization of psychometric data of a specific psychopathology, namely eating disorders (EDs) via a systematic review of the methods presented in literature carried out following the PRISMA guidelines (Fig 1; [1]). EDs are severe psychiatric syndromes defined by abnormal eating behaviors that negatively affect a person’s physical or mental health [2]. They are believed to result from and be sustained by sociocultural, psychological, and biological factors. Anorexia nervosa (AN), bulimia nervosa (BN), and binge eating disorder (BED) are the primary diagnoses associated with ED.

In the last century, the paradigm that best got ahead in Western medicine has been the “disease model” [3], according to which all symptoms a person exhibits result from a common cause or latent entity (namely, the underlying disease) that should therefore be targeted by an effective treatment to obtain, as a consequence, the lessening of all the deriving symptoms [4,5].

**Fig 2:**
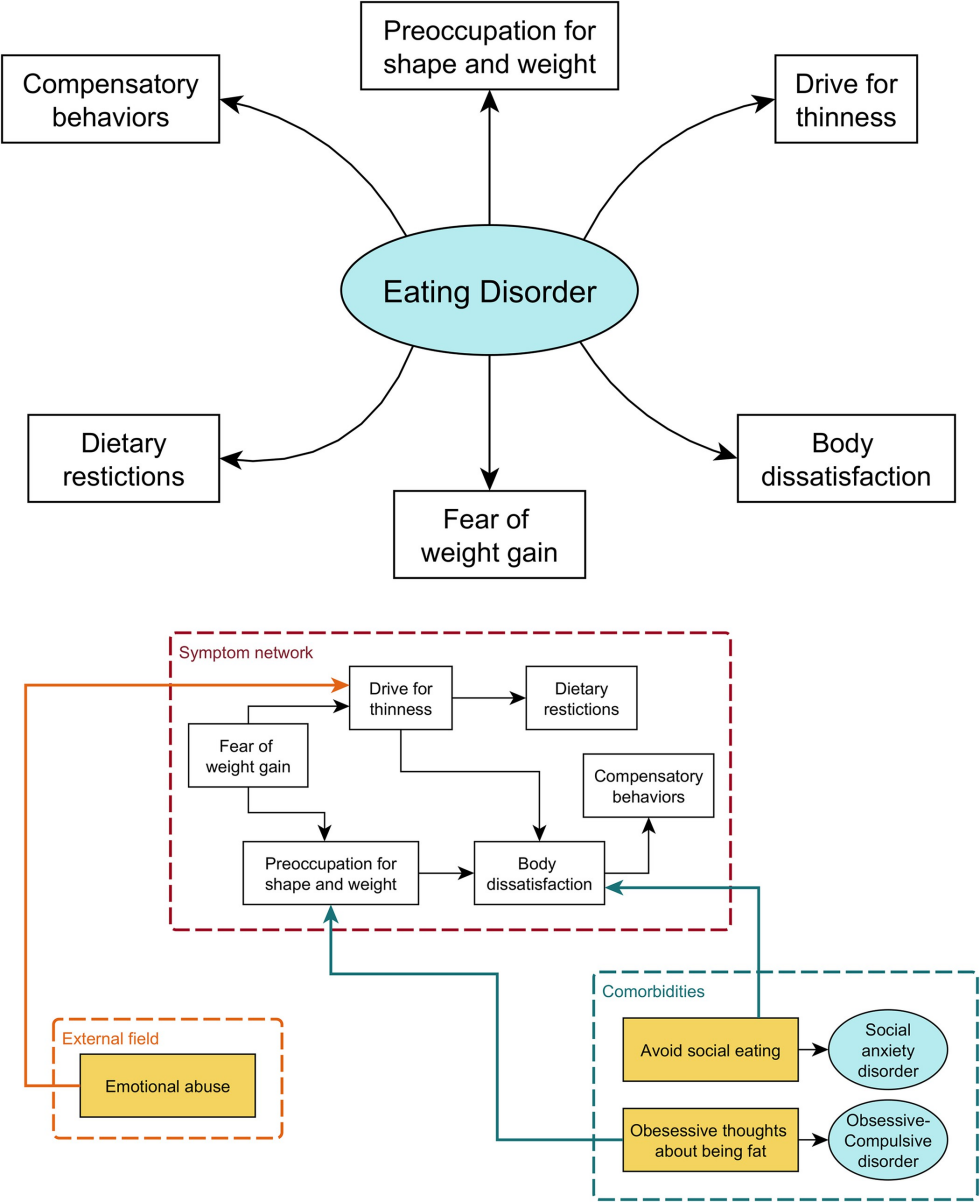
2a and 2b. Comparison between Factor and Network Model. Schematic representation of factor model (a) and network model (b) of eating disorders (simplified). While in the first case symptoms (white rectangles) are considered manifestations of some common underlying factor (e.g., the eating disorder psychopathology [cyan ellipse]), according to the network model symptoms are conceptualized as mutually interacting and reciprocally reinforcing elements of a complex network where ED-specific symptoms (white rectangles in the red dashed box) mutually influence non-specific ones (yellow rectangles), such as external events (orange dashed box) or comorbidities (cyan ellipses in the blue dashed box). Hence, symptoms are seen as causally active components of the mental disorder instead of passive receptors of its causal influence.

Unfortunately, in contrast with general medicine, in most mental disorders the identification of common pathogenic pathways has proven elusive [3,5–7], given that they cannot be diagnosed independently of their symptoms [3]. Therefore, the need of conceptualizing in an alternative way the relation between symptoms and disorders arose in the twenty-first century and led to the delineation of the network theory of psychopathology, an innovative approach that inspired an exponentially increasing number of empirical publications in the past two decades, especially after the seminal article by Borsboom and Cramer [3] was published. Differently from the disease model, in the network model, symptoms are conceptualized as mutually interacting and reciprocally reinforcing elements of a complex network, i.e., causally active components of the mental disorder instead of passive receptors of its causal influence (see **Fig 2**).

### The network approach to psychopathology: A theoretical framework

The central idea behind the network approach to psychopathology is that mental disorders arise from casual interactions between symptoms, where causality must be interpreted in the sense of the interventionist theory, according to which the relation between two symptoms is causal if there exists a possible (natural or experimental) intervention on one of them that changes the probability distribution of the other, independently of the how the causal relationships are triggered [6].

In order to represent and study these symptom-symptom interactions, a network structure can be used, a so-called *symptom network*. In the scientific setting, the term *network* refers to a mathematical structure called *graph*, which consists of a set of nodes connected by links, or, in more formal terms, an ordered pair *G = (V*, *E)* where *V* is the set of vertices (or nodes), i.e., the system’s components, and *E* is the set of edges (or links), i.e., the interactions between them. If the edges have no direction, thus indicating a two-way relationship, then the graph is said to be *undirected*; otherwise, if the edges are given a specific direction, that is, they can only be traversed in a single direction, then the graph is called *directed*. Moreover, each edge can also be given a number called *weight*, that represents a quantification of its strength or cost or capacity, according to the different context. In this case, the graph is said to be *weighted* to distinguish it from the *unweighted* type [8]. The arrangement of the network’s elements is called *topology*.

Although no distinction is usually made, the terminology “graph”, “vertex”, “edge” refers more precisely to the mathematical representation of the system, whereas “network”, “node”, “link” is more common in reference to real systems such as physical, biological, social, and economical systems. The network approach has proven successful and useful in a number of fields, from social sciences to economics, informatics, ecology, epidemics, biology, and medicine, among others (cfr. [9–14]). In the specific case of symptom networks, nodes encode symptoms and edges stand for causal influence between pairs of symptoms.

There might also be conditions that can change the state of symptoms from the outside of the psychopathology network, for example adverse life events, abnormal brain functioning and inflammation, among others; all together, these constitute the *external field* of the symptom network [6]. Another crucial property is the existence of partially overlapping syndromic clusters or bridge symptoms, that is, symptoms that are associated with multiple disorders and thus are part of symptom networks corresponding to different psychopathologies. This feature allows for an immediate explanation of the high level of comorbidity that characterizes mental disorders [5–7,15].

An ultimate facet that needs to be considered to complete the theoretical framework of network analysis applied to psychopathology is the proven existence (in most psychopathology networks) of a phenomenon called *hysteresis*, which is a fundamental indicator of the dynamics of the system and consists in the dependence of any state of the system to its history [16]. In other words, once a system has been activated by an external event, the subsequent fading of that event will not necessarily deactivate that symptom in case there exist connections with other symptoms that are strong enough to make the reactions provoked by the triggering event (i.e., the activated symptoms system) self-sustaining [6]. As proven by Cramer [16], the hysteresis effect becomes more pronounced as the connectivity of a network increases. In fact, what has been noticed is that in weakly connected networks, even though significant triggering events can cause strong reactions, once the event is over, the system will gradually recover and return to its asymptomatic state. In this sense, a weakly connected network is said to be *resilient*, as opposed to the *vulnerable* disposition of the strongly connected ones [6].

Following these observations, Borsboom [6] proposed new definitions of *mental health* as the stable state of a weakly connected network and *mental disorder* as an alternative stable state of a strongly connected network which is separated from the healthy state by hysteresis.

At this point, it is important to underlie that the conceptualization of the network approach to mental disorders should not be regarded as a theoretical finding only. Indeed, it has remarkable implications for the diagnosis and treatment systems as well [6,17].

### The psychometric network analysis workflow

The term *psychometric network analysis* is used to describe the combined procedure of network estimation, network description and network stability analysis, which together build the bulk of the methodology used in network approaches to multivariate psychometric data [3,15,18–20].

**Fig 3:**
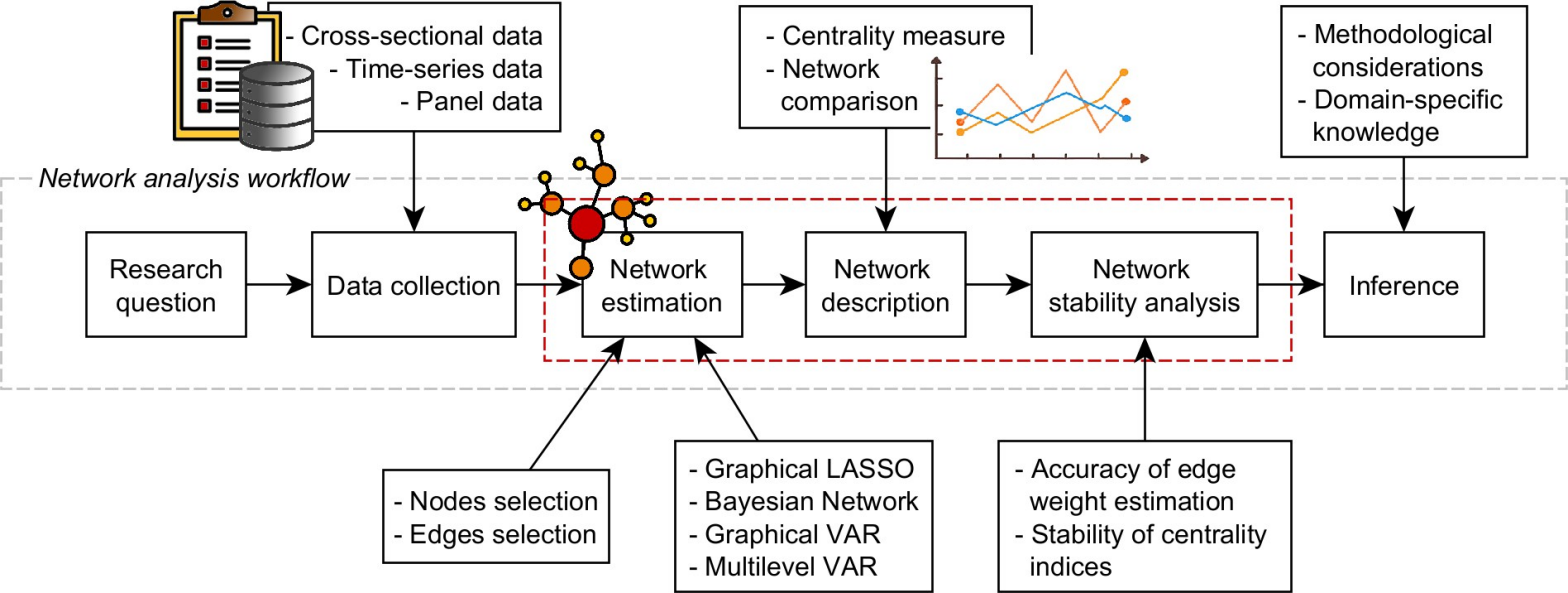
Psychometric network analysis workflow. Scheme of the typical workflow of psychometric network analysis. Once the research question has been defined (also according to the availability of data), the main steps to be performed are: 1. Network estimation, that is, construction of the network. 2. Network description, that is, identification of important symptoms. 3. Network stability analysis, that is, assessment of the robustness of results. Together, these will allow to infer significant interpretation that should be employed in clinical treatment.

The complete workflow (**Fig 3**) typically starts with a specific research question, according to which a suitable data collection scheme is chosen. Usually, experimental data is given in the form of either a cross-sectional, time-series or panel design. Although the subsequent procedures are generic statistical ones and thus apply to input variables of any kind, in this context psychometric variables usually consist of responses to questionnaire items, symptom ratings and cognitive test scores together with other possible personal or psychological indicators [18].

Once enough data are available, the network estimation step can be carried out with the aim of approximating the values of links between pairs of nodes (i.e., the causal influence of one onto the other) and building an appropriate network structure at the system level. Depending on the peculiarities of the data, different statistical methods can be employed: the most frequent approach is that of assessing the edge parameters as conditional associations between variables to estimate the corresponding *Pairwise Markov Random Field* (PMRF), but the alternative strategy of *Bayesian network* estimation has been successfully employed as well [5]. Importantly, this step also encompasses the process of *node selection* and *edge selection*, the latter via general statistical methods such as fit indices, null-hypothesis testing, or cross-validation procedures.

The result of this step is generally a nontrivial topological structure which becomes the main subject of the *network description* phase, whose aim is to give a complete characterization of the symptom network with a particular focus on its most important nodes. Here, “importance” has to be intended as how a node is interconnected with the other nodes of the network and is commonly assessed by different centrality measures [5], that is, scalar values assigned to each node within a graph in order to assess their significance based on certain definitions of importance. In general, the tools of network analysis are employed to estimate network density and connectivity through global topological properties, node centrality through local topological properties and more fine-grained structural patterns such as communities and motifs (i.e., mesoscopic level [21]).

Next, it is fundamental to evaluate the stability and robustness of the estimated network and of the centrality indices. In fact, the estimation error and the sampling variation need to be considered in order to not obtain misleading results [22–26]. Altogether, the methods used to assess the accuracy of the estimated parameters and their ability to replicate in a different dataset constitute the *network stability analysis* [25].

Finally, the psychometric network analysis approach comes to an end with proper inferences which require taking into account both substantive domain knowledge and methodological considerations about the stability and robustness of the estimated network [18].

The clinical implications of such findings concern both the diagnosis and treatment systems. With respect to diagnosis, the network approach suggests clinicians to follow a two-step process according to which they should firstly identify the exhibited symptoms and then the network interactions that sustain them, which however is actually not very different from the current DSM diagnostic practice [6]; the novelty introduced by this approach is that it can help recognize the most important symptoms as well as other psychological traits that are not included in the DSM criteria [27], such as feeling ineffective and social insecurity in case of EDs. As for the treatment of the diagnosed disorder, the network perspective suggests clinicians to apply techniques that can change or manipulate the network, in particular that are capable of: changing the state of one or more symptoms, modifying the external field by removing triggering events or manipulating the network structure by altering the connections among symptoms [6]

### Aim of the systematic review

The aim of this study is to collect and review the existing literature on psychometric network analysis of EDs. To the best of our knowledge, three other reviews centered on the application of this approach to EDs have already been published between 2018 and 2021 [28–30]. As already pointed out in [30], although giving important insights into the framework and potentialities of the network research on EDs, Levinson et al., 2018 [28] and Smith et al., 2018 [29] suffer from the limitation of reviewing only a restricted number of studies (3 and 5, respectively). On the other hand, the most recent review paper [30] examines a wider range of articles (i.e., 25) and precisely discusses their results, but leaves out an accurate analysis of the methodologies employed by the researchers. In addition, as already pointed out by the authors, a noteworthy limitation of this latter review is that no study based on longitudinal data was taken into consideration. With this systematic review, we intend to update and broaden the results of such seminal reviews with an even larger and wide-ranging sample of studies, in particular by focusing on the potentialities and limitations of the available methodologies in the field of psychometric network analysis.

## Methods

To ensure a standardized review procedure, the Preferred Reporting Items for Systematic Reviews and Meta-analyses (PRISMA) 2020 statement [1] was followed. No protocol was a-priori registered. As this is a systematic review of published literature, ethical approval was not sought.

We also followed guidelines derived by tools such as ROBIS (available at https://www.bristol.ac.uk/population-health-sciences/projects/robis/robis-tool/), aimed at evaluating and identifying concerns with the review process and at judging risks of bias. We carefully assessed the whole review process, i.e., each of the following steps in a sequential manner: study eligibility criteria; identification and selection of studies; data/method collection and study evaluation; synthesis and new findings. All steps were judged to have low or no concern, and consequently the review was assessed as having a low risk of bias.

The articles included in this study were extracted from the databases Scopus, PubMed and PsycInfo in February 2022 by means of a query aimed at retrieving all published articles containing the simultaneous occurrence of terms related to network analysis and EDs; more precisely, we searched for: (“eating disorder* OR “anorexia” OR “bulimia”) AND “network analysis” AND NOT “social network” in either the title, abstract or keywords of the articles indexed in the selected databases. Searching the three databases yielded a set of 235 articles, including 135 duplicates, and was further narrowed down to a number of 57 papers by considering the following exclusion criteria: (1) studies not based on psychometric data, (2) studies with aims different from the investigation of EDs, and (3) review articles. All detailed information about each included article is reported in **Table 1**. The PRISMA flow chart corresponding to the present methodology is shown in **Fig 1**.

**Table 1:** Summary of the information of major interest of the 57 studies included in the systematic review.

Ref.	Research question*	Sample size	Data type	Gender	Age range	Diagnosis**	Scale***	Network type
Aloi et al., 2021 [31]	C	155	Cross sectional	86.5% F	18–65	BED	EDI-2 + others	GLASSO
Beauchamp et al., 2021 [32]	B	144	Cross sectional	67.6% F	20–85	NED	NEQ + EDE + others	GLASSO
Bronstein et al., 2022 [33]	C, D	310	Cross sectional	59% F	18+	non clinical	EPSI, DRS + others	GLASSO
Brown et al., 2020 [34]	C	428	Cross sectional	95.26% F	μ = 21.70	AN, BN, BED, OSFED	EDE-Q	GLASSO
Calugi et al., 2020 [35]	E	547 (adolescents) + 724 (adults)	Cross sectional	96.3% (adol.) 95.9% (adul.)	12–19 (adol.) 20–61 (adul.)	AN	EDE-Q	GLASSO
Calugi et al., 2021 [36]	G	214	Panel data	95.3% F	16+	AN	EDE-Q	GLASSO
Cascino et al., 2019 [37]	C	84	Cross sectional	F	μ = 28.76 (AN-BP) μ = 26.33 (AN-R)	AN	EDI-2	GLASSO
Christian et al., 2020 [38]	E	29902 (EPSI group) 32219 (EDE-Q group)	Cross sectional	94% (EPSI) 96.5% (EDE-Q)	11–85 (EPSI) 17–79 (EDE-Q)	non clinical	EDE-Q + EPSI	GLASSO
Christian et al., 2021 [39]	E	6850	Cross sectional	83.4% F	13–79	AN, OSFED, BN, BED, non clinical	EDE-Q + EPSI	GLASSO
Cusack et al., 2021 [40]	D	1072	Cross sectional	F	μ = 19.47	non clinical	EDE-Q + EPSI + others	GLASSO
De Paoli et al., 2020 [41]	D	753	Cross sectional	81.5% F	μ = 22.36	AN, BN, BED, OSFED	EDI-3 + others	GLASSO
de Vos et al., 2021 [42]	C	905	Cross sectional		17+	AN, BN, BED, OSFED	EDE-Q + others	GLASSO
DuBois et al., 2017 [43]	A	194	Cross sectional	88.6%	10+	AN, BN, BED, ARFID, OSFED, Rumination	EDE-Q + EPSI	GLASSO
Elliott et al., 2020 [44]	D, G	142 (baseline) 119 (6m follow-up) 113 (12m follow-up) 105 (24m follow-up)	Panel data	139 F	18–60	AN, EDNOS	EDE	GLASSO
Forbush et al., 2016 [45]	B	143	Cross sectional	77.6% F	18–55	AN, BN, BED, OSFED	EPSI	Association and concentration networks
Forrest et al., 2018 [46]	A, B	1081	Cross sectional	F	13–69	AN, BN	EDE-Q	GGM
Forrest et al., 2019 [47]	B	1150	Cross sectional	M	μ = 27.06	non clinical	EDE-Q + others	FGL
Forrest et al., 2019 [48]	D	296	Cross sectional	95.8% F	14–60	AN, BN, BED, ARFID, OSFED	EDE-Q + others	GLASSO
Giles et al., 2022 [49]	D	320	Cross sectional	F	14–70	AN, BN	EDI-2 + EATATE	GLASSO
Goldschmidt et al., 2018 [50]	A	636	Cross sectional	90.3%	6–18	AN, BN, OSFED	EDE	GLASSO
Hagan et al., 2021 [51]	G	409	Cross sectional	67%	12–18	AN	EDE + others	GLASSO
Hilbert et al., 2020 [52]	G	178	Panel data	-	18+	BED	EDE + others	GLASSO
Kenny et al., 2021 [53]	D	4421	Cross sectional	42.6% M	10–15	non clinical	EDE-Q + others	GLASSO
Kerr-Gaffney et al., 2020 [54]	D	101	Cross sectional	95% F	18–55	AN, ASD	EDE-Q + others	GLASSO
Kinkel-Ram et al., 2021 [55]	D	352	Cross sectional	60% M	19–72	non clinical	EDE-Q + others	GLASSO
Levinson et al., 2017 [56]	D	196	Cross sectional	95.4% F	μ = 28.2	BN	EDE + others	GLASSO
Levinson et al., 2018] 65]	D	2215	Cross sectional	87% F	14–83	ED, SAD	EDI-2 + EDE-Q + others	GLASSO
Levinson et al., 2018 [57]	F	64	Panel data	97% F	14–41	AN, Atypical AN, BN, OSFED	EDDS + EDI-2 + EPSI + EDE-Q + EMA	mlVAR + graphicalVAR
Levinson et al., 2020 [58]	F	1272	Panel data	F	13–55	non clinical	EDDI	mlVAR + graphicalVAR
Levinson et al., 2021 [59]	F	34	Panel data	91.2% F	20–57	AN, BN, BED, Atypical	EMA	graphicalVAR
Levinson et al., 2021 [60]	C, D	120	Cross sectional	55% F	22–65	non clinical	EPSI + others	GLASSO
Mares et al., 2022 [61]	A	336	Cross sectional	94% F	12–68	AN, BN, BED	EDE-Q	
Martini et al., 2021 [62]	C	139 (P = patients) 121 (C = control)	Cross sectional	94.96% F (P) 91.74% F (C)	16–55	AN	EDI-2 + others	FGL
Meier et al., 2020 [63]	D	303	Cross sectional	84.8% F	18–79	AN, BN, EDNOS	EDE-Q + others	GLASSO
Monteleone et al., 2020 [64]	C	77	Cross sectional	F	16–55	AN, BN	EDE-Q + others	GLASSO
Monteleone et al., 2022 [65]	A, C	325	Cross sectional	-	18+	BN, BED	EDI-2 + others	GLASSO
Monteleone et al., 2021 [66]	G	*Patients vs TAU*: 88, 99 (baseline) 71, 75 (end of treat.) 58, 63 (6m follow-up) 53, 63 (12m follow-up)	Panel data	96.8%	16+	AN, OSFED	EDE-Q + others	MGM
Monteleone et al., 2019 [67]	C	228	Cross sectional	F	18+	AN, Atypical AN, BN	EDI-2 + others	GLASSO
Monteleone et al., 2019 [68]	C	405	Cross sectional	92% F	9–18	AN	EDI-3 + others	GLASSO
Olatunji et al., 2018 [69]	G	5193	Panel data	F	12–68	AN, BN, EDNOS	EDI-2 + others	GLASSO
Perez et al., 2021 [70]	E	818	Cross sectional	F	18–78	non clinical	EDE-Q	GLASSO
Perko et al., 2019 [71]	E	1343	Cross sectional	50% F	18+	AN, BN, BED, OSFED	EDE-Q + EPSI	GLASSO
Ralph-Nearman et al., 2021 [72]	C	267	Cross sectional	94.8% F	14–61	AN, Atypical AN	EDDS + others	GLASSO
Rodgers et al., 2019 [73]	C	327	Cross sectional	-	15+	AN, BN, BED, EDNOS	EDE-Q + others	Bayesian Network
Rodgers et al., 2018 [74]	B	251	Cross sectional	F	18+	non clinical	EDI-3	GLASSO
Sahlan et al., 2021 [75]	D	1749	Cross sectional	43.4% M	12–54	non clinical	EDE-Q + others	GLASSO
Sahlan et al., 2021 [76]	D	730	Cross sectional	55.5% M	9–13	non clinical	ChEAT	GLASSO
Schlegl et al., 2021 [77]	E	2535	Cross sectional	F	mixed	AN, BN	EDI-2	FGL
Smith et al., 2020 [78]	C	538 (clinical outpatients) 166 (suicide attempt) 238 (ED)	Cross sectional	60% (clinical outpatients) 71% (suicide attempt) 96% (ED)	μ = 26.55 (clinical outpatients) μ = 28.74 (suicide attempt) μ = 17.61 (ED)	AN, BN, BED, EDNOS, OSFED	EDI-3 + others	FGL
Smith et al., 2019 [79]	D, G	446	Panel data	84% F	16–64	AN, BN, EDNOS	EDE-Q + others	GLASSO
Solmi et al., 2018 [80]	A, C	2068	Cross sectional	96.6% F	μ = 23.13 (AN) μ = 26.06 (BN) μ = 35.31 (BED)	AN, BN, BED	EDI-1 + others	GLASSO
Solmi et al., 2019 [81]	A, C	955	Cross sectional	-	μ = 25.69 (AN-BP) μ = 21.81 (AN-R)	AN	EDI-1 + others	GLASSO
Vanzhula et al., 2019 [82]	D, E	158 (clinical) 300 (non clinical)	Cross sectional	95.6% F (clinical) 100% F (non clinical)	14–59 (clinical) 17–23 (non-clinical)	AN, BN, BED, OSFED	EDDS + EDE-Q + others	GLASSO
Vanzhula et al., 2021 [83]	D	1696	Cross sectional	94% F	18+	AN, BN, BED, OSFED	EDE-Q + others	GLASSO
Vervaet et al., 2021 [84]	C	7969	Cross sectional	95.8% F	13–67	AN, BN, BED, EDNOS	EDI-2 + others	GLASSO
Wang et al., 2019 [85]	B	788	Cross sectional	74.2% F	18–65	BED	EDE	GLASSO
Wong et al., 2021 [86]	C	196	Cross sectional	94.8% F	15–66	AN, BN, BED, ARFID	EDE-Q + others	GLASSO

* Legend of the field “Research question”: A = validation of the transdiagnostic model of EDs; B = estimation of the symptom network of EDs and identification of the core symptoms; C = identification and interaction with nonspecific ED symptoms; D = assessment of psychiatric comorbidities; E = comparison of symptom network; F = estimation of intraindividual networks; G = assessment of treatment outcome.

** Acronyms used within the column “Diagnosis”, listed in order of appearance in the text: EDI-2 (Eating Disorder Inventory, 2nd revision [87]), NEQ (Night Eating Questionnaire [88]), EDE (Eating Disorder Examination [89]), EPSI (Eating Pathology Symptoms Inventory [90]), DRS (Dietary Restriction Screener [91]), EDE-Q (Eating Disorder Examination Questionnaire [92]), EDI-3 (Eating Disorder Inventory, 3rd revision [93]), EATATE [94], EDDS (Eating Disorder Diagnostic Scale, [95]), EDDI (Eating Disorder Diagnostic Interview [96]), ChEAT (Children’s Eating Attitudes Test [97]), and EDI-1 (Eating Disorder Inventory, [98]).

*** The column “Scale” explicitly lists only the psychometric questionnaires for the assessment and evaluation of EDs. The term “others” indicates that other tests assessing non-specific ED symptoms have been used.

## Results

In the following, we analyzed a large sample of network-based studies that exploit psychometric data related to ED. Specifically, we first introduce each step of the (general) psychometric network analysis workflow and then describe and compare the results reported in the articles under review.

### Research question

In line with the application to other mental disorders, various research goals can be identified among the existing literature about network approaches to EDs, namely:

- validation of the transdiagnostic model of eating disorders by comparing network characteristics across ED diagnoses [43,46,50,61,65,80,81];
- estimation of the symptom network of EDs and identification of the core symptoms [32,45–47,74,85];
- identification and interaction with nonspecific ED symptoms (i.e., the external field) like general psychiatric symptoms, personality traits and other clinical variables [68,80,81], embodiment dimensions [37], childhood maltreatment [60,65,67,73], mentalizing and empathy [64], vulnerability factors [84], suicidal thoughts and behaviors [78], perfectionism and interoceptive sensibility [62], affective and metacognitive symptoms [31,86], interoceptive awareness [34], sleep disturbance [72], well-being domains (49), inflexible and biased social interpretations, socioemotional functioning [33];
- assessment of psychiatric comorbidities such as depression and anxiety [33,44,53,56,75,79], posttraumatic stress disorder [60,82], social anxiety disorder [76,99], obsessive- compulsive disorder [49,55,63,83], trait anxiety disorder [48], autism spectrum disorder [54], borderline personality disorder [41], and alcohol misuse [40];
- comparison of estimated network structures among clinical and nonclinical [82], ethnic minority women [70], men and women [71], across developmental stages [35,38,77], and across different duration of illness [39];
- characterization of the dynamic structure of systems and evaluation of intraindividual networks [57–59];
- assessment of treatment outcome [36,44,51,52,66,69,79].

### Collection of psychometric data

The accomplishment of the above research goals relies on the successful collection of datasets having specific peculiarities, since this will determine the possibility of estimating certain types of networks. The most typical starting point for this kind of analysis is clearly the selection of appropriate psychometric assessment tools, mainly self-report questionnaires and structured clinical interviews [5]. Depending on the sample size and the sampling frequency, three types of data environments can be identified among the current practice of network approaches to psychopathology, namely cross-sectional data, time-series data, and panel or longitudinal data.

*Cross-sectional data* has been the first type of data used in this field and is definitely the most mentioned across the existing literature (e.g., it was used in 48 papers out of 57 in our sample). It is particularly suitable for the estimation of group-level networks, since it provides variable measures taken at a single time point in a large sample. Importantly, the associations between variables are built upon differences among individuals and for such a reason a lot of caution should be taken when inferences about single patients are made, since the very strict conditions under which a structure of intraindividual variation can be deduced from the analogous structure of interindividual variation are seldom met in psychological processes [100,101]. In a cross-sectional dataset, rows can be reasonably assumed to be independent, therefore the corresponding PMRF can be directly estimated from it.

*Time-series* and *panel data* have been introduced in the psychometric network modeling in order to address two main limitations of cross-sectional data: the lack of clear understanding of individual networks and the inability to capture the dynamic features of psychopathology [5]. Both data environments are characterized by datasets where variables are measured at multiple time points, with the difference that time-series data focuses on one single individual, whereas panel data consist of observations of multiple individuals. Given a time-series dataset, one can estimate two different structures: a directed temporal network of vector autoregressive coefficients where links describe associations between variables through time, and an undirected contemporaneous network where links describe instead the association between variables after the temporal effects have been removed. In case of panel data, a third structure can be estimated, namely a between-subject network, where links indicate the conditional associations between the long-term averages of the time series between people [18].

In line with other experimental studies, the applications to EDs mostly move from cross-sectional data. Nevertheless, few exceptions are worth mentioning. Firstly, Levinson and coworkers in three different papers [57–59] used panel data to estimate interindividual networks (temporal, contemporaneous, and between-subject), as well as intra-individual networks (temporal and contemporaneous) for some of the patients in the sample. Other relevant studies aimed at assessing the treatment efficacy by applying statistical techniques [34,36,38,44,51,52,66,79].

Data vary in terms of other features as well. Among the articles under review, 50 out of 57 described their sample as being composed of a great majority of female participants. After all, the fact that EDs are much more common in women than in men is broadly known and documented [102], with reasons usually attributed to social pressure [103], adolescent turbulence, poor body concept, and role confusion [104]. Just one study involved only male participants [47], while few other papers reported a more heterogeneous (mostly nonclinical) sample with male participants within the range of 40–60% [33,53,55,60,71,75,76].

Moreover, in 60% of the cases, participants are reported as clinical, either inpatients or outpatients. Among these, three studies involved users of the Recovery Record [105] smartphone application [38,39,71]. Exceptions consist in mixed samples involving nonclinical patients, such as school or college students, and three case studies based on datasets collected through the crowdsourcing marketplace Amazon Mechanical Turk, (MTurk; [47,55,60]).

Nearly all papers focus on the most common ED diagnosis, namely Anorexia Nervosa (AN) and Bulimia Nervosa (BN). However, some of them also present results concerning secondary EDs, in particular binge-eating disorder [52,85], and night eating disorders [32].

Various psychometric assessment questionnaires were used as tools for data collection. For the evaluation of ED specific symptoms, the most widely used tests were the Eating Disorder Inventory (EDI; [87,93,98], the Eating Disorder Examination Questionnaire (EDE-Q; [92]), and the Eating Pathology Symptom Inventory (EPSI; [90]). For the assessment of general psychological factors other tests were also used, for example the Symptom Check‐List 90 (SCL-90; [106,107]), and the Beck Depression Inventory (BDI-II; [108]). Note that, among the cited psychometric tests, the only one that has been designed to assess both ED specific symptoms as well as other general integrative psychological constructs is EDI.

### Methods for network estimation and reconstruction

Once the data has been collected, the next fundamental point is determining the variables of interest, that is, the nodes of the network. Instead of considering the totality of the items included in the questionnaires, a common practice is that of reducing their number in an effort to produce more accurate results by avoiding redundant (i.e., collinear) variables. The final nodes do not generally comprise all the items of the questionnaires. Instead, they are chosen in either one of the following ways, namely by taking into account just some special item aggregates such as questionnaires’ subscales, by employing the *goldbricker()* function of the *R* package *networktools* [109], which compares correlations between variables and identifies the collinear ones, or by combining the latter with a further theoretically driven selection of items.

### Cross-sectional networks

The types of networks that can be estimated depend on what kind of data is available. In case of cross-sectional data, the main solutions are association networks, concentration (or partial correlation) graphs, regularized partial correlation networks, and Bayesian networks, where the first three types are undirected, weighted and can all be estimated with the *qgraph* [110] *R* package, while the last one is direct, either weighted or unweighted, and can be obtained with the help of the *bnlearn* [111] *R* package.

*Association networks* are the most basic types of networks that can be estimated from cross-sectional data. Edges correspond to zero-order correlations between symptoms, indicating the probability of their co-occurrence. For example, the *qgraph() R* function with input parameter graph = “cor” will compute an association network by estimating zero-order correlations between each pair of variables through the Pearson coefficient *r*.

Consider a set of *p* variables *X* = (*X*_1_,…,*X*_*p*_), each described by *n* observation, that is, $X_{i}=(x_{i}^{\left(1\right)},\ldots ,x_{i}^{\left(n\right)})\;\forall i$. Given paired data $\left\{\left(x_{i}^{\left(1\right)},x_{j}^{\left(1\right)}),\ldots ,(x_{i}^{\left(n\right)},x_{j}^{\left(n\right)}\right)\right\}$, the Pearson coefficient is defined as:

$$r_{X_{i}X_{j}}=\frac{\sum_{k=1}^{n}(x_{i}^{\left(k\right)}-{\bar{X}}_{i})(x_{j}^{\left(k\right)}-{\bar{X}}_{j})}{\sqrt{\sum_{k=1}^{n}{\left(x_{i}^{\left(k\right)}-\bar{X_{i}}\right)}^{2}\sqrt{\sum_{k=1}^{n}{\left(x_{j}^{\left(k\right)}-{\bar{X}}_{j}\right)}^{2}}}}$$

where *n* is the sample size, $x_{i}^{\left(k\right)}$ and $y_{i}^{\left(k\right)}$ are two sample points indexed with *k*, and ${\bar{X}}_{i},{\bar{X}}_{j}$ are the sample means of the variables *X*_*j*_ and *X*_*j*_.

Association networks have two main limitations: first, they do not give any information about the direction of causal relationships, and second, they do not discern true relations from spurious ones and from those caused by the influence of other nodes [5].

*Concentration networks* solve the second of these limitations by estimating edges as partial correlations between symptoms after adjusting for the influence of all other nodes in the networks; only the edges whose value is above a fixed threshold are then kept. Formally, the partial correlation between two variables *X* and *Y* given a set of *n* controlling variables *Z* ={*z*_1_,…,*z*_*n*_} is written as *Corr*(*X*_*i*_,*X*_*j*_|*Z*) and is given by the correlation between the residuals *e*_*X*_ and *e*_*Y*_ resulting from the linear regression of *X* with *Z* and of *Y* with *Z*, respectively. A network where each edge corresponds to the partial correlation between the connected nodes can be estimated through the *qgraph()* function by setting the parameter graph = “concentration”.

When dealing with *p*-multivariate data $X=(X_{1},\ldots ,X_{p})∼N(\mu ,\Sigma )$ all information needed to compute the partial correlation coefficients is encoded in the variance-covariance matrix Σ. In fact, once its inverse is defined (i.e., the so-called precision matrix *K*), one can directly apply the following relationship to recover the partial correlation coefficients:

$$\mathrm{Corr}\left(X_{i},X_{j}|X_{-(i,j)}\right)=-\frac{k_{ij}}{\sqrt{k_{ii}k_{jj}}}$$

where *k*_*ij*_ denotes the element in row *i* and column *j* of the precision matrix *K* = ∑^−1^ and *X*_−(*i*,*j*)_ denotes the set of variables without *X*_*i*_ and *X*_*j*_. These coefficients can be graphically displayed in a weighted undirected network where each node corresponds to a variable and edges between them are given by the partial correlation coefficients. If the *ij*^th^ component of ∑^−1^ is zero, then the variables *X*_*i*_ and *X*_*j*_ are conditionally independent, given the other variables, and no edge will be traced between them. This model is a type of PMRF and it is called *Gaussian Graphical Model*, shortly GGM [24]. Forbush et al. [45] gave an example of an ED symptom network estimated as an association graph and also proposed the corresponding concentration network in the supplementary material of the same paper.

When the number of variables to estimate is high, it has been suggested that a more appropriate model to use is the *regularized partial correlation network* [112], obtainable running the *gqraph() R* function with input parameter graph =“glasso”. The result is similar to a concentration graph in the fact that edges indicate partial correlation between nodes, however it has the relevant difference of including the implementation of an L1 regularization technique called *graphical LASSO* (least absolute shrinkage and selection operator; [113]) which allows for the shrinking of all small partial correlations to zero. This procedure returns a sparse network that parsimoniously accounts for the covariance among nodes, in the sense that only the edges that are most robust and most likely to reflect real associations are kept [5]. Formally, the graphical LASSO gives an estimation of the precision matrix *K* = ∑^−1^ by solving the optimization problem of maximize the penalized log-likelihood

$$\underset{K}{\max}\{\log\;\det\;K-\mathrm{trace}(SK)-\lambda ||K|{|}_{1}\}$$

over nonnegative definite matrices *K*, where *S* is the empirical covariance matrix of *X*, λ is a nonnegative tuning parameter and ||*K*||_1_ denotes the $l_{1}$-norm, that is, the sum of the absolute values of the elements of ∑^−1^. Hence, the higher the λ value, the more *K*_*ij*_ will be set to zero.

Clearly, if λ is too low, then too many spurious edges risk being included (i.e., yielding a high number of false positives), whereas if λ is too high, then the risk is to remove relevant connections (false negatives). Hence, the λ parameter needs to be tuned. The best model (i.e., the most likely to maximize the number of “true” edges while minimizing the spurious ones) is then identified through the Extended Bayesian Information Criterion (EBIC; [114]) for multiple λ values and then choosing the model with lowest EBIC score. Let *P* be a subset of {1,…,*p*} and let *υ* = |*P*| be the cardinality of this subset, then the best λ is chosen to be the one that maximizes

$${\mathrm{EBIC}}_{\gamma }(P)=-2L_{\Sigma }(X)+\upsilon \;\log\;n+2\gamma \;\log\;p$$

where *L*_Σ_(*X*) is the log-likelihood *L*_Σ_(*X*) = −log det *K*−trace(*SK*) [114,115].

It has been suggested by Williams and Rast [116] that the graphical LASSO gives an accurate representation of data only when the number of nodes vastly exceeds the number of cases; if not, a non-regularized network should be preferred. Nevertheless, almost all of the articles under review based on cross-sectional data employ the graphical LASSO to estimate the symptom network.

Notably, in Monteleone et al. [66] the network structure of the cross-sectional data corresponding to each time period of the analysis is estimated via a Mixed Graphical Model (MGM), an extension of GGM that allows the integration of variables following two or more different data distribution [117]. In the specific case of this study, MGM was chosen in favor of GLASSO to include the treatment group as a binary variable into the model and thus assess its effect with respect to the treatment as usual (TAU) control group.

*Bayesian networks* attempt to discern causality by representing data as directed acyclic graphs (DAGs) where arrows indicate the direction of predictions and, possibly, causality [7]. DAGs depict the joint probability distribution of the variables and can thus be decomposed into the conditional distribution of each node given its parent. Importantly, dependence relations should not be confused with temporal antecedence, which cannot be derived from cross-sectional data in any way. What restrains Bayesian networks from widespread application is the existence of strict assumptions about data that it is pretty difficult to find in psychological analysis settings according to clinical observations [5]. The first condition is that all relevant causal variables should be included in the system. Second, the causal Markov Condition [7] must be met. Third, the probability distributions of certain variables might not be unrestricted. And fourth, it might not be easy to choose the best model among all possible ones [7]. Moreover, one additional assumption is suggested by the definition itself of DAGs, namely that all loops of any length are prohibited. A Bayesian network can be estimated from multivariate data through the *bnlearn* R package [111].

In a paper that analyzes the relationship between EDs and childhood abuse, Rodgers et al. [73] followed this Bayesian approach in two steps: they first wrote down a “blacklist” of forbidden edges to limit the investigation to patterns of symptom relationships that made both conceptual and clinical sense; next, they estimated the DAG through the hill-climbing algorithm. This is an iterative machine-learning process that starts with an arbitrary network structure and tries to improve it by making incremental changes to the network (e.g., adding, removing, or reversing edges). At each step, the Bayesian Information Criterion (BIC) is computed, and the change is kept if and only if it results in a lower BIC. This process continues until no further improvements can be found and the network model that represents the best fit for the interactive structure of the ED psychopathology is returned.

Diverging from the other studies, Bronstein et al. [33] employed a combined procedure to test different hypotheses. In particular, they used the exploratory causal discovery algorithm *Greedy Fast Causal Inference* (GFCI; [118]) to investigate potential causal pathways involving ED symptoms, biased and inflexible interpretations, and socioemotional functioning. GFCI takes as input a dataset of continuous variables (e.g., psychometric data) and outputs a graphical model called *Partial Ancestral Graph* (PAG; [119]), which is a representation of a set of Bayesian Networks that cannot be distinguished by the algorithm. Notably, GFCI is characterized by the ability of detecting latent confounders (i.e., an unmeasured variable that casually influences two or more measured variables); this information is conveyed by different edge types in the generated PAG.

### Joint estimation of cross-sectional networks

In many of the studies under review, multiple networks were estimated with the specific aim of comparing their structure in various populations. As it will be later explained in more details, most often this task is accomplished by first estimating each network separately and only later some pivotal test statistics are computed to highlight global and local topological differences.

However, when the observations in a dataset consist of several distinct classes, it is also possible to adopt a recently developed technique called *Joint Graphical Lasso* (JGL), which allows for jointly estimating multiple graphical models corresponding to distinct but related conditions [120]. In particular, the JGL fits GGMs on data with the same variables observed on different classes but differs in the estimation of uncoupled GGMs from independent samples in the fact that, besides the lasso penalty on density, the regularized optimization problem for the evaluation of the precision matrices of each class includes an additional penalty term that is specifically written to foster similarity between groups.

Suppose we are given *K* datasets Y^(1)^,…,*Y*^(*K*)^,*K*≥2, where each Y^(*k*)^ is a *n*_*k*_×*p* matrix consisting of *n*_*k*_ observations on a set of *p* features common to all *K* datasets. We assume that all the observations $\sum_{k=1}^{K}n_{k}$ are independent and that, within each dataset, $Y^{\left(k\right)}∼N(\mu_{k},\Sigma_{k})$. Then one can define the empirical covariance matrix for *Y*^(*k*)^ as $S^{\left(k\right)}=\frac{1}{n_{k}}{\left(Y^{\left(k\right)}\right)}^{T}Y^{\left(k\right)}$. Danaher et al. [120] proposed to estimate the precision matrices (∑^(1)^)^−1^,…,(∑^(*K*)^)^−1^ by maximizing the penalized log-likelihood

$$\max_{\theta }\{\overset{K}{\underset{k=1}{\sum }}n_{k}(\log\;\det\theta^{\left(k\right)}-\mathrm{trace}(S^{\left(k\right)}\theta^{\left(k\right)}))-P(\{\theta \})\}$$

where *θ*^(1)^,…,*θ*^(*k*)^ are assumed to be positive definite and *P*({*θ*}) denotes a convex penalty function chosen to encourage precision matrices to share certain characteristics (e.g., the locations or values of the nonzero elements or the sparsity).

Depending on the explicit definition of the penalty function, JGLs are classified as Fused Graphical Lasso (FGL) and Group Graphical Lasso (GGL). The former encourages shared edge values across classes, whereas the latter only encourages a similar pattern of sparsity across all precision matrices. Hence, the FGL results in a stronger form of similarity [120]. The penalty of the FGL has the form

$$P(\{\theta \})=\lambda_{1}\overset{K}{\underset{k=1}{\sum }}\underset{i\neq j}{\sum }|\theta_{ij}^{\left(k\right)}|+\lambda_{2}\underset{k<k^{\prime }}{\sum }\underset{i,j}{\sum }|\theta_{ij}^{\left(k\right)}-\theta_{ij}^{\left(k^{\prime }\right)}|$$

where *λ*_1_ and *λ*_2_ are nonnegative tuning parameters, the first controlling the`$l_{1}$-penalties applied to each off-diagonal element of the *K* precision matrices, the second those applied to the differences between corresponding elements of each pair of precision matrices. Hence, large values of *λ*_1_ will increase the sparsity of the precision matrices (just like in graphical LASSO), while large values of *λ*_2_ will cause many elements to be identical across classes.

Both types of JGL have already been implemented in the *R* package *EstimateGroupNetwork* [121], which also include methods for the automatic tuning parameter selection. A final consideration about JGL is that its network estimations cannot behave worse than independent GGMs [120–122]. In fact, when the tuning procedure selects a value of the corresponding tuning parameter equal or very close to zero, independent GGMs are estimated via typical graphical lasso. Therefore, JGL cannot hide differences nor inflate similarities across groups.

The JGL has been applied in the EDs research as well. Schlegl et al. [77] used the FGL with *k*-fold cross-validation for parameter selection to estimate four different networks, one for each of the following groups: adolescents with AN, adults with AN, adolescents with BN, and adults with BN. Similarly, Smith et al. [78] used the FGL to estimate and compare distinct networks from samples corresponding to either of the following groups: outpatients without ED diagnosis, outpatients with a lifetime attempt of suicide, and people with a current ED diagnosis. Martini et al. [62] employed FGL to compare a sample of AN patients with a control group. Finally, [47] jointly estimated and compared the network structures of two nonclinical samples of men with and without core ED symptoms.

### Longitudinal and personalized networks

Time-series data allow for the estimation of personalized networks of two different kinds. *Temporal networks* incorporate consecutive temporal effects among symptoms by representing arrowhead edges pointing from one node to the other (or to itself in case of self-loops) when the first predicts the second in the next window of measurement. Edges are also weighted according to the regression parameters [123,124]. Temporal networks are commonly estimated as lag-1 Vector-Autoregression (VAR) models [125], which consist of a set of regression equations on the given variables; these are all treated as endogenous, so they act as both outcome and predicted variables. The relations assessed through this method can also be interpreted in terms of Granger causality [126] by stating that, whenever an arrow from a time-varying symptom X to another time-varying symptom Y is found, then X *Granger-causes* Y, meaning that predictions of the value of Y based on its own past values and on the past values of X are better than predictions of Y based only on Y’s own past values. Clearly, Granger causality should not be erroneously understood as pure causality. Rather, Granger causality gives evidence of temporal prediction and thus it can be potentially indicative of causality in the sense that, although the existence of a causal relationship would imply the observation of a temporal prediction, the opposite is not true, since temporal links may also arise for other reasons. In addition, some temporal predictions can also be missed because of lack of statistical power or insufficient sized lag interval [124].

The residuals of the temporal VAR model are used to compute the so-called *contemporaneous network*, which can be estimated as a GGM model, with edges representing the partial correlation obtained after controlling for both temporal effects and all other variables in the same window of measurement [124]. Together, the modeling framework including the estimation of both temporal and contemporaneous networks from a given dataset is called *graphical* VAR or GVAR [127]. It can be computed with the help of the R package *graphicalVAR* [128], which allows for both regularized and unregularized estimations [123].

When time-series of multiple subjects are available, it is possible to gain more insight into the network structure at the group-level by applying the *multilevel-VAR model*. For a given population, the average network parameters are called “fixed effects”, whereas the person-specific deviations from these fixed effects are called “random effects”. Random effects can be used to estimate intraindividual networks (temporal and contemporaneous) and to investigate interindividual differences [129]. Fixed effects can be instead used to uncover information about average intraindividual effects. More generally, if a multivariate normal is assumed for all parameters, then estimating the GVAR model on longitudinal data allows for the decomposition of the variance into three different structures, namely temporal networks, contemporaneous networks and *between-subjects networks* [123], where the latter is a GGM that allows for the examination of between-mean relationships for all individuals of the dataset. Estimation methods for multilevel-VAR models have been implemented in the *R* package *mlVAR* [128], which in particular permits to choose among different estimation procedures, such as sequential univariate multi-level estimation, multivariate Bayesian estimation, and fixed effects estimation.

In the context of the studies about EDs, Levinson et al. [57] first conducted a pilot study in which they collected longitudinal data from *N* = 66 participants by asking them to complete an Ecological Momentary Assessment (EMA) survey [130,131] on ED cognitions and behaviors across one week. Using the *graphicalVAR* and *mlVAR* packages, they then estimated intraindividual networks to identify which symptoms maintain EDs within each individual as well as group-level networks, namely temporal, contemporaneous and between-subject networks. Later, they repeated an analogous study on a different dataset composed of longitudinal data of *N* = 1272 participants with the additional goal of comparing adolescents and adults network structures [58]. More recently, another study was conducted to exclusively estimate the individual networks of *N* = 34 participants with the aim of identifying and discussing a range of target symptoms for personalized ED treatment [59].

### Approaches and tools for network description

The analytical phase that follows the estimation of the network structure from data is designated to the investigation and interpretation of specific characteristics exhibited by either single nodes or groups of nodes jointly. Although a first visual inspection can give some general clues, many numerical methods can be employed to gain deeper insights.

#### Centrality measures in GGMs

Depending on the type of network estimated, different measures can be used to investigate the role of each symptom in the network. In the case of undirected weighted networks, Opsahl et al. [132] proposed a generalization to weighted networks (see **Table 2**) of the most common measures of node centrality that were originally designed by Freeman [133] for binary networks, namely: degree, strength, closeness, and betweenness centrality.

**Table 2:** Definition and interpretation of common centrality measures.

Centrality Measure	Formulation	Interpretation
**Degree:** number of edges connected to a node	$C_{D}(v)=\sum_{u\in V\backslash \{v\}}e_{vu}$ where *e*_*vu*_ is an edge between *u* and *v*	Higher values indicate higher centrality of that node in the network, that is, a central node is one with many connections. It is useful mainly in unweighted graphs.
**Strength (S):** sum of the absolute weights (e.g., partial correlation coefficients) of the edges connected to a node	$C_{S}(v)=\sum_{u\in V\backslash \{v\}}{|w}_{vu}|$ where |*w*_*vu*_| is the absolute weight between *u* and *v*	It represents the likelihood that activation of a certain symptom will induce the activation of other direct symptoms [5]. Just like the degree centrality, strength only accounts only for paths of unitary length.
**Closeness (C):** inverse of the sum of the (minimum) distance of a node to all the other nodes in the network. It can be normalized as the average length of the weighted shortest paths to all the other nodes	$C_{C}(v)={\left[\sum_{u\in V\backslash \{v\}}d(u,v)\right]}^{-1}$ where *d*(*u*,*v*) is the distance between *u* and *v*	It indicates the index of expected time until arrival of something flowing through the network. In simple words, a node with high closeness is one that is close, on average, to other nodes. Hence, nodes with low closeness centrality are likely to be sooner influenced by changes in the network.
**Betweenness (B):** number of the geodesics between any two other nodes that pass through a given node	$C_{B}(v)=\frac{g_{uz}(v)}{g_{uz}}$, where *g*_*uz*_ is the number of binary shortest paths between two nodes, and *g*_*uz*_(*v*) is the number of those paths that go through node *v*	This measure plays an important role in the assessment of comorbidities, since symptoms with high betweenness centrality usually serve as bridge symptoms; when activated, they are likely to spread to both syndromic clusters [5]. In other words, a high-betweenness node is one that acts as a bridge in the communication with other nodes.
**(One-Step) Expected Influence (EI):** it computes the strength of a node by considering both positive and negative correlations	$EI(v)=\sum_{u\in V\backslash \{v\}}w_{vu}$ where *w*_*vu*_ is the weight of the edge between *u* and *v*	It exhibits the same performance of strength when the network only contains positive weights, whereas it outperforms strength as the number of negative weights increases. It has been proven that EI is a better predictor of declines in the severity of symptoms over time. It is particularly useful when the aim is to identify target symptoms for therapeutic deactivation since these can only be identified through negative correlations.

This table describes the centrality measures that are most commonly used in the analysis of undirected weighted networks, as formalized by Opsahl et al. [132]. The last row describes the (one-step) expected influence centrality [134]. For each measure, its definition, mathematical formulation and interpretation are given.

However, all the measures above only consider weights in absolute value with the consequence that two nodes may have same centrality but opposing effects on the rest of the network. For example, an increase in Node A can cause an increase in Node B (positive influence) with the same strength that an increase in Node C causes a decrease in Node D (negative influence). In this case, Node A and C will have the same centrality index but an opposite effect. Moreover, a node with a similar number of strong positive and negative edges may have little to no overall network activation impact, that is, it might be highly central without being highly influential. Hence, a new centrality metric called *expected influence* (EI) has been introduced to consider both negative and positive edges (see **Table 2**, last row; [134]).

As argued by Bringmann et al. [135], betweenness and closeness centrality do not seem to be especially suitable as measures of node importance. Hence, results about these metrics should be interpreted with care. As an additional proof about this fact, many articles reported betweenness and closeness centrality as not satisfying the minimum stability results and did not include their estimation in the network description [31,36,46,64,74,79,82]. Others decided instead to take these measures into account but did not assess their stability [45,69].

Throughout the large number of studies reviewed, few symptoms appeared among the most central ones across heterogeneous samples and estimation techniques. Among these: shape and weight overvaluation, body dissatisfaction, drive for thinness, ineffectiveness (i.e., feeling of inadequacy, insecurity, worthlessness and having no control over one’s life), and lack of interoceptive awareness (i.e., ability to identify, access, understand, and respond properly to the patterns of internal signal). For a complete list refer to **Table 3**.

**Table 3:** Central symptoms in psychopathology networks.

Symptom	Centrality Measure	References
Shape and weight preoccupation and overvaluation, body dissatisfaction	S and EI	(41,50–55,58,61,63,68,70,72,74,84,95,96,98)
Fear of weight gain	S and EI	(41,42,51,53,58,59,63,64,68,70,74,81,84,95)
Drive for thinness	S, EI, C and B	(39,43,45,47,51,54,59,63,66,70,79–81,85,86,89,91,94,95)
Ineffectiveness	S, EI, C and B	(49,73,76,78,80,83,86,89,92,94,97)
Lack of interoceptive awareness	S, C, and B	(43,47,76,78,80,92,94,97)
Social insecurity, in particular avoidance of social eating	S, EI, C and B	(36,43,62,73,80,83,98)
Dietary restriction	S and EI	(41,42,55,58,59,72,75,84)
Depressed mood	S and EI	(33,36,49,84,92,94)
Binge-eating	S	(44,45,65,68,82,95)

This table summarizes the symptoms (left column) that were most often identified as central in the psychopathology networks built from psychometric data concerning either specific or nonspecific ED symptoms. The central column indicates the metric used (S = strength, EI = expected influence, C = closeness, and B = betweenness). The column on the right contains the list of papers that find the symptom as a result of their analysis.

Diverging from the other studies, 3 papers also assessed the importance of nodes using *key players* analysis. Specifically, they identified the nodes that, when removed, would result in a maximally disconnected residual network. In other words, a treatment targeting these key nodes is expected to slow the cascade of symptoms through the ED network. Analyzing the symptom network of a sample of participants with mixed ED diagnoses, Forbush et al. [45] identified its key players to be: *people encouraged me to eat more*, *need to exercise nearly every day*, and *try to avoid foods with high calorie content*. Perko et al. [71] employed this metric to assess sex differences in ED symptoms, but they found that the key players were, in both cases, items related restricting dieting and binge eating. More recently, Liebman et al. [60] explored the associations between posttraumatic stress disorder and ED symptom in presence of at least one experience of childhood abuse and found that the key players of the network were: *purging*, *negative alterations in cognitions and mood*, and *hyperarousal*.

#### Interpretation and measures of importance in Bayesian networks

When interpreting a Bayesian network visualized as a DAG, node importance cannot be evaluated through the above-mentioned centrality measures for undirected graphs. Rather, Rodgers et al. [73] proposed to assess this property based on the relative contribution of each symptom to the overall model fit of the Bayesian network. In particular, they evaluated the *scaled contribution of each symptom to BIC* for three different DAGs: one estimated from the full sample, one from the subsample of participants who experience childhood abuse in addition to having received an ED diagnosis, and one for the subsample of those that only suffer from an ED. The result was that in the first and third group the most important symptoms were *overvaluation of shape and weight*, *depressed mood* and *eating large amounts of food*, whereas in the second group a different pattern of relationships among symptoms emerged, with *depressed mood*, *dietary restraint*, *self-induced vomiting*, and *driven exercise* being the most important driving symptoms of the disorder. The dissimilarity found was consistent with the concept of *maltreated ecophenotype* [136], according to which distinct subtypes of a given disorder may be developed as a consequence of abuse and trauma. This hypothesis has been also confirmed by other experimental studies on different psychiatric disorders including EDs [137].

#### Understanding comorbidities between EDs and other psychopathologies

With the specific aim of identifying bridge symptoms, four network statistics have been developed and implemented in the *R* package *networktools*; importantly, since they are not specific to the type of network estimated, they can be readily applied to intraindividual networks, group-level networks, and other networks different from the psychopathology ones [138]. They are defined as follows:

- *Bridge strength* (BS), that is, the sum of the absolute value of every edge which connects a given symptom to symptoms belonging to other disorders. *Bridge in-strength* and *bridge out-strength* can be analogously defined in directed networks by considering only the subset of edges that are, respectively, directed toward the node or issued from a node.
- *Bridge expected influence* (BEI), which is defined just like the bridge strength but without taking the absolute value. In directed networks, only edges issuing from a node are summed.
- *Bridge Closeness* (BC), that is, the average distance from a given node to all nodes outside of its own disorder.
- *Bridge betweenness* (BB), that is, the number of times a given node lies on the shortest path between any two nodes belonging to two distinct disorders (including the one it belongs to).

Refer to **Table 4** for the list of the bridge symptoms between EDs and other psychopathologies that have been assessed through the metrics defined above in some of the papers under review.

**Table 4:** Bridge symptoms between eating disorders and other psychopathologies.

Comorbidity	Bridge Symptoms	References
Trait anxiety	Avoidance of social eating (ED) Lacking self-confidence (anxiety)	[48]
Social anxiety disorders	Social eating and drinking	[99]
	Nervousness focused on appearance	[33,99]
	Concern over being judged	[76]
Autism spectrum disorder	Poor self-confidence Concerns over eating around others Concerns over others seeing one’s body	[54]
Posttraumatic stress disorders	Binge-eating (ED) Irritability (PTSD) Desire for a flat stomach (ED) Concentration problems (PTSD)	[82] (clinical sample)
	Food‐related concentration difficulties (ED), Weight‐ and shape related concentration Difficulties (ED), Irritability (PTSD) Loss of interest (PTSD)	[82] (nonclinical sample)
	Reexperiencing (PTSD) Cognitive restraint (ED)	[60]
Obsessive- compulsive disorder	Interpersonal distrust	[49]
	Desire to lose weight Sudden thoughts about being fat	[55]
	Difficulty controlling obsessions	[63]
	Difficulties controlling thoughts	[83]
Borderline personality disorder	Abandonment Emotion dysregulation Attachment avoidance	[41]
Depression	Feelings of worthlessness Having a negative reaction to wanting to weigh oneself weekly Not wanting to eat in social situations	[44,53,56,75,79]
Alcohol misuse	Drinking in the morning Purging Guilt about drinking	[40]

This table lists, for each of the analyzed comorbidity with EDs (left column), the symptoms with highest bridge EI (center column) as reported in the reference papers (right column).

#### Assessing the role of the external field in the development and maintenance of Eds

Interactions between ED specific symptoms and various elements of the external field have also been investigated. Few studies used the bridge centrality measures to accomplish this task. Monteleone et al. [67,137] chose instead to adopt a different approach to explore the psychological pathways through which childhood maltreatment (CM) experiences promote the development of ED core symptoms. Namely, they first selected few variables from items and scores of different psychometric questionnaires in order to build symptom networks for each ED diagnosis, then the shortest path between any CM and ED node was computed using Dijkstra’s algorithm, and finally they used mediation analysis to confirm the mediation role of the symptoms included in the shortest pathways from CM to ED specific symptoms. All the other studies considered all variables as a single community and chose to determine the core symptoms using the centrality measures in their classical form with the specific aim of verifying whether EDs are mainly maintained by ED specific symptoms or rather by nonspecific ones. The results achieved throughout the papers under review are summarized in **Table 5**.

**Table 5:** Interaction between eating disorders and the external field.

External Field	Scale*	Bridge Symptoms	Central symptoms	References
**Interoceptive awareness**	EDE-Q MAIA	*Highest BEI*: Feeling unsafe in one’s body		[34]
**Mentalizing and empathy**	EDE-Q DASS-21 MASC EAT-R	*Highest BS*: Restraint eating Emotional state inference	*Highest S*: Under-mentalizing (MASC) Over mentalizing (MASC) Cognitive mental state Inference (MASC) Shape concern (EDE-Q)	[64]
**Interoceptive awareness**	EDE-Q MAIA	*Highest BEI*: Feeling unsafe in one’s body		[34]
**Perfectionism, interoceptive sensibility**	EDI-2 MAIA FMPS	*Highest BEI*: Perfectionistic evaluative concerns Mistrust in body sensations		[62]
**Suicidal thoughts and behaviors**		Interoceptive deficits Pain tolerance		[78] (current ED diagnosis and patients with a lifetime suicide attempt)
		Feeling inadequate		[78] (outpatients)
**Inflexible and biased social interpretations socioemotional functioning**	EPSI PHQ SIAS/SPS …	*Highest BEI*: Psychomotor agitation/ retardation (PHQ), Excessive/depressed appetite (PHQ) People staring while you walk down the street (SPS)	*Highest EI and exploratory causal discovery*: Social anxiety Depression Negative social exchange	[33]
**Affective states**	SCID-5 PANAS EDE-Q	*Highest BEI*: Guilt about eating	*Highest EI*: Guilt about eating Weight-based judgment of self	[86]
	EDI-2 MSAS DERS …		*Highest S*: Impaired self-monitoring metacognition (MSAS) Difficulties in impulse control (DERS)	[31]
**General psychiatric symptoms**	EDI-3 MASC YSR …	*Highest S*: Depression symptoms Personal alienation Asceticism	*Highest BEI*: Depression symptoms Personal alienation Low self-esteem Interoceptive deficits	[68]
	EDI-1 SCL-90 TPQ		*Highest S*: Ineffectiveness Depression Anxiety	[80,81] (across all diagnoses)
**Well-being domains**	EDE-Q OQ-45 MHC-SF	*Highest BS*: Self‐acceptance Environmental mastery Feeling depressed	*Highest S*: Psychological well‐being	[42]
**Embodiment disturbances**	EDI-2 IDEA		*Highest S*, *B and C*: Interoceptive awareness (EDI) Feeling extraneous from one’s own body (IDEA)	[37]
**Childhood maltreatment (CM)**	EDI-2 CTQ	*Shortest paths between each childhood trauma node and ED core symptoms + Mediation analyses*: AN-R: emotional abuse à interoceptive awareness AN-BP & BN: emotional abuse à ineffectiveness and interoceptive awareness BED: emotional abuse à impulsivity, ineffectiveness and interoceptive		[67,137]
	EDE-Q BDI CTQ		*Bayesian score*: Loss of control eating Depressed mood	[73]
	EPSI PCL-5 …	*Highest BS*: Reexperiencing (PCL-5) Cognitive restraint		[60]
**Sleep- disturbance**	EDE-Q BDI-II …	*Highest BEI*: Feeling tired or fatigued Loss of energy Physical anxiety concerns	*Highest S*: Judgment based on shape Restriction Feeling tired or fatigued	[72]
**Depression, anxiety, and vulnerability factors**	EDI-2 YSQ FMPS …		*Highest EI*: Over-vigilance and inhibition (YSQ) Interoceptive awareness Ineffectiveness (EDI-2) Impaired Autonomy and Performance (YSQ)	[84]
	EDE-Q BDI-2 RSES	*Highest BEI*: Feeling like a failure	*Highest S*: Desiring to lose weight Feeling like a failure	[75]

This table summarizes the results achieved throughout the papers under review concerning the role of the external field in the development and maintenance of EDs. For each specific external factor, many details are given, namely:Tthe psychometric assessment tool used to assess it, both bridge and central symptoms identified in the psychopathology network, and the reference paper.

** Acronyms used within the column “Diagnosis”, listed in order of appearance in the text: MAIA (Multidimensional Assessment of Interoceptive Awareness [139]), DASS-21 (Depression, Anxiety, and Stress Scales—Short Version [140,141], MASC (Movie for the Assessment of Social Cognition [142]), EAT-R (Empathic Accuracy Task—Revised [143,144], FMPS (Frost Multidimensional Perfectionism Scale [145]), PHQ-9 (Physicia’s Health Questionnaire-Depression Module [146]), SIAS/SPS (Social Interaction Anxiety Scale / Social Phobia Scale-Short Forms [147]), SCID-5 (Structured Clinical Interview for DSM-5 [148]), PANAS (Positive and Negative Affect Schedule [149]), MSAS (Metacognition Self-Assessment Scale [150]), DERS (Difficulties in Emotion Regulation Scale [151], YSR (Youth Self Report [152]), TPQ (Tridimensional Personality Questionnaire [153]), OQ-45 (Outcome Questionnaire [154], MHC-SF (Mental Health Continuum Short Form [155]), IDEA (IDentity and EAting disorders [156]), CTQ (Childhood Trauma Questionnaire [157]), PCL-5 (PTSD Checklist for DSM-5 [158]), YSQ (Young Schema Questionnaire [159]), and RSES (Rosenberg Self-Esteem Scale [160]).

#### Longitudinal studies

All longitudinal studies that used the *graphicalVAR* and *mlVAR* for network estimation also computed node centrality through the following measures: strength for between-subject and contemporaneous networks, in-strength and out-strength for temporal networks. In the latter case, that specific choice of metrics was taken to underlie the different impact of incoming against outgoing edges. More precisely, *In-strength* is given by the sum of links pointing towards the node and indicates how much information that node is receiving from directly connected nodes. Instead, *out-strength* is given by the sum of links pointing from one node to all the other and indicates how much information that node is sending to directly connected nodes. This distinction has the precious advantage of suggesting which nodes (i.e., those with highest out-strength), have the potentiality of having downstream effects on other symptoms if treated [59].

As for the group-level temporal networks, the symptoms with highest in-strength centrality were *desire to be thin*, *body checking* [57], *fasting*, *fear of weight gain* and *feeling fat* [58,59]. Those with highest out-strength centrality were *exercise, binge eating [57]*, *feeling fat* and *fear of weight gain* [58]. The strongest symptoms in the contemporaneous and between-subject group-level networks were *desire to be thin* [57], and *feeling fat [58]*. Among the individual networks, nodes with highest in-strength centrality in the temporal networks were *overvaluation of weight and shape*, *fear of weight gain*, and *body dissatisfaction* [58]; while those with highest out-strength were: *exercise, fear of weight gain, overvaluation of weight and shape [58]*, and *body dissatisfaction* [59]. Finally, the strongest symptoms in the individual contemporaneous networks were *thinking about dieting*, *binge eating* [57], *fear of weight gain [58]*, *body dissatisfaction* and *drive for thinness* [59].

#### Pre- to post-treatment studies

Few studies have been conducted to compare pre- and post-treatment network models. In particular, they all estimate and compare GGM networks at each time point (mainly at admission and discharge, in few cases also at follow-up) and few studies also assess the prognostic value of the most central nodes at baseline through linear or logistic regression. One of them [44] first computed zero-order correlations between each symptom at baseline and three outcome measures (i.e., treatment recovery status, clinical impairment, and posttreatment BMI) and then tested whether these prognostic values were associated with the expected influence of symptoms at baseline via linear regression. The results of this study show that EI values remained constant across all time points, with the strongest nodes being *feeling fat*, *fear of weight gain*, *discomfort seeing one’s own body*, *dissatisfaction with weight*, and *a strong desire to lose weight*. The authors also found that more severe symptom levels were associated with a lower possibility of recovery, higher clinical impairment and higher BMI. Finally, they observed that centrality of symptoms at baseline was significantly associated with prognostic values for both recovery status and clinical impairment. Similarly, Hagan et al. [51] found that pretreatment central symptoms in adolescents with AN significantly predicted early response but did not predict remission. A third study [69] also found that the most central symptoms, namely *interoceptive awareness* and *ineffectiveness*, did not change after treatment. The authors also used multiple regression to test whether the identified core symptoms predicted outcomes (BMI, depression, and anxiety) at discharge. Their hypothesis was confirmed for BMI and depression but not for anxiety. Finally, Brown et al. [34] showed that stronger *desire to lose weight* at admission was associated with lower likelihood of achieving remission at discharge.

Many other studies only estimated and compared the centrality of symptoms at different time points. A change in the role of certain symptoms was found, with the strongest at baseline being *fearing weight gain, dietary rules [36]*, *eating disorder-related impairment*, *self-esteem* and *shape concern* [52], and the strongest at discharge being *dietary restraint [36,52]*, *food preoccupation*, *feelings of fatness* and *discomfort seeing its own body* [36]. Smith et al. [79] only computed centrality measures of the admission network, finding that the strongest symptoms were: *shape and weight-related concentration difficulties*, *general concentration difficulties*, *guilt about eating*, *desire to lose weight*, and *nervousness*.

Finally, the study of Monteleone et al. [66], explicitly designed to assess the clinical change promoted by TAU enhanced by RecoveryMANTRA compared to TAU alone, did not compute the centrality of symptoms as it was out of scope, but it focused instead on the differential improvement in symptom strengths between the two samples at each time point (from baseline to 12-months follow-up). In particular, they found that RecoveryMANTRA was associated with a direct effect on few symptoms (i.e., anxiety, shape concern and restraint but not on motivation, stress and depression as hypothesized) only at the end of the intervention.but not at follow-up. Furthermore, they computed the predictability of symptoms in terms of explained variance [161] and found an increase in predictability of the network from baseline to 12 months follow-up, suggesting that treatment indeed promoted changes in the associations between symptoms.

### Network stability analysis

Network stability analysis has been conducted in most of the papers under review, especially in those published after 2018, when Epskamp and his colleagues Borsboom and Fried [24] proposed precise guidelines for this task. In particular, they suggested specific methods to assess the robustness of the model at three distinct levels: accuracy of the estimated edge weights, stability of the order of centrality indices, and difference between specific edge weights or centrality indices.

#### Accuracy of edge weights

The accuracy of edge weights can be evaluated by constructing intervals that reflect the sensitivity of edge weight estimates to sampling error, such as confidence intervals (CIs), credibility intervals and bootstrapped intervals [18]. When handling ordinal data as in the current study, it has been suggested to derive the (1-α) CIs via nonparametric bootstrap at a given confidence level, for example, α = 0.05 [24]. The narrower the CIs, the more likely is that the estimated edge weight is close to its real value, since in 95% of the cases such a CI will contain the true value of the parameter. Although large CIs can result in a poor accuracy for centrality indices, they do not influence the presence of an edge, nor its sign, as these properties are already assessed by LASSO. Moreover, it should be noticed that since we use regularization to estimate the network structure, all edge weight estimates are biased towards zero and, consequently, all sampling distributions are biased towards zero as well, just like CIs are not centered around the true unbiased parameter value anymore. This implies that, when interpreting the quantiles of the bootstrapped sampling distribution, if they overlap with zero it could be that the corresponding CI does not overlap with zero, while if they do not overlap with zero, then also the corresponding CI does not overlap with zero. In other words, CIs should not be interpreted as significance tests to zero, but only to show the accuracy of edge weight estimates by evaluating the size of CIs and to compare edges to one another by checking if the corresponding CIs do not overlap; if so, then we can conclude that they significantly differ at the given significance level, in the other case, then we cannot infer the contrary since they might still significantly differ [24].

#### Stability of centrality indices

As noticed by Epskamp and colleagues [24], the same bootstrap technique cannot be used to construct true CIs around the centrality indices. As an alternative, they proposed to investigate the stability of the order of centrality indices based on subsets of the data, that is, by comparing the order of centrality indices after re-estimating the network with fewer cases or nodes. When this is done for various proportions of cases to drop, then one can also assess the correlation between the original centrality indices and those obtained from subsets. If this correlation keeps stable after dropping a considerable proportion of the cases (e.g., 10%), then the interpretations of centralities can be considered stable. This method has been called *case-dropping subset bootstrap*. Exploiting this technique, a quantification of the stability of a centrality index can be given by the *correlation stability coefficient* (CS-coefficient), a measure representing the maximum proportion of cases that can be dropped, such that the correlation between original centrality indices and that of networks built on subsets with 95% probability is still equal or higher than a given value which is set to 0.7 by default. As a cutoff score for interpreting an estimated centrality index as stable, Epskamp and colleagues [24] suggested that the CS-coefficient value should be above 0.5 or in any case not below 0.25. As already mentioned, this cutoff was not reached by the CS-coefficient of closeness and betweenness centrality in almost all cases. On the other side, strength and expected influence always attained pretty good values, usually significantly above the threshold.

#### Methodological differences in edge weights and centralities

The last technique proposed by Epskamp [24] is the *bootstrapped difference test*, which is a null-hypothesis test used to assess whether the edge weights or centralities differ from one another. This is accomplished by considering the difference between the two bootstrap values of edge weights or centrality indices under study and constructing a bootstrapped CI around those difference scores. If zero is not in the bootstrapped CI, then the null hypothesis is rejected, meaning that there is evidence that two values differ from one-another. On the other hand, it should be noticed that not rejecting the null-hypothesis is not sufficient for inferring that the null-hypothesis is true. Finally, Epskamp et al. [24] emphasized and warned about the fact that this technique does not take into account any correction for multiple testing, since applying Bonferroni correction is not feasible in practice in this context. As a consequence, as the number of performed significance tests increases, the probability of finding several significant results purely by chance (Type 1 error) also increases.

#### Other approaches for stability estimation

The above-mentioned stability techniques do not apply to Bayesian networks. As an alternative, Rodgers et al. [73] quantified the arc strength, that is, the degree of confidence it is possible to have when interpreting specific pathways, through a bootstrapping procedure introduced by Friedman et al. [162,163] and implemented in the *bnlearn R* package. The general idea behind this procedure is that we should be more confident on features that would still be induced when we perturb the data. Therefore, in a nonparametric bootstrap setting, one first generates perturbations by re-sampling with replacement from the given dataset and then examines how many of the perturbed structures exhibit the feature under study that, in this specific case, corresponds to the presence and direction of each edge. Their relative frequency across the bootstrapped samples gives an estimation of each arc strength [164]. Rodgers et al. [73] reported an average frequency of 84% concerning the presence of edges correctly identified across bootstrapped samples, and an average frequency of 63% for their direction. The arc *fear of gaining weight → cognitive restraint* had the highest presence frequency of 99%, while the arc *preoccupation with eating and body image → depressed mood* had the highest direction frequency of 88%.

### Common methods for network comparison

Many of the studies under review aimed at comparing network structures across different populations. A specific tool to accomplish this task, namely the *Network Comparison Test* (NCT; [17]) has been devised and implemented in the *R* package *NetworkComparisonTest* [165]. Compared to other statistical tests, the NCT overcomes the usual assumption of normality and possible improper null hypothesis that are unsuitable for the regularized parameters that results after GGM estimation with LASSO regularization, the most typical method employed in the network approach to psychopathology.

The NCT is a 2-tailed permutation test in which the difference between two groups is calculated repeatedly for randomly regrouped individuals. It consists of three steps: first, the network structure is estimated for both groups using the original data and the metric of interest is calculated; second, data is permuted iteratively to rearrange group memberships, networks are then re-estimated, and metrics are calculated based on permuted data to create a reference distribution; finally, the significance of the observed test statistic is evaluated by comparing it to the reference distribution. In particular, the *p*-value equals the proportion of test statistics that are at least as extreme as the observed test statistic. Thus, the null hypothesis that the two networks under comparison are the same can be rejected if the latter is larger than expected (i.e., *p*-value < 0.05).

As for the test statistics that can be used to compare networks, Van Borkulo et al. [165] proposed three metrics that represent both global and local differences, namely invariance of network structure and global strength for the former case, and invariance of edge strength for the latter (see **Table 6**).

**Table 6:** Network comparison.

Metric	Definition	Interpretation
**Invariant network structure (M-test)**	It evaluates the null hypothesis that all edges are equal. Specifically, the Chebyshev norm of the vector containing all differences of corresponding edge weights in the two networks is calculated. $M(N_{1},N_{2})={\left\|w^{1}-w^{2}\right\|}_{\infty }={max}_{ij}(\left|w_{ij}^{1}-w_{ij}^{2}\right|)$	If this is higher than some threshold d, then at least one of the differences is larger than d. On the contrary, if the maximum of all differences is not significant, then none of the differences is significant, meaning that the null hypothesis cannot be rejected.
**Invariant edge strength** **(E-test)**	It evaluates the null hypothesis that a pair of corresponding edges have equal weight $E(w_{ij}^{1},w_{ij}^{2})=|w_{ij}^{1}-w_{ij}^{2}|$	It represents the likelihood that activation of a certain symptom will induce the activation of other direct symptoms [7]. Just like the degree centrality, strength only accounts only for paths of unitary length.
**Invariant global strength** **(S-test)**	It evaluates the null hypothesis that the overall level of connectivity (i.e., global strength) is the same across subpopulations. $S(N_{1},N_{2})=\sum_{i=1}^{p}\sum_{j>1}\left|w_{ij}^{1}\right|-\sum_{i=1}^{p}\sum_{j>1}\left|w_{ij}^{2}\right|$	If this difference is not close to zero, then one can conclude that the symptom network of one group is more densely connected then the other, for example, because of the presence of risk factors.

Definition and interpretation of the three test statistics introduced by van Borkulo et al. [165] to compare a pair of networks based on their global and local differences.

The above test statistics have already been used in several studies about EDs. Significant results concerning the invariant network structure have been found in various comparisons, in particular: clinical versus nonclinical subsamples of networks assessing comorbidity between EDs and different features of social anxiety disorder [99]; admission against discharge network [36]; adolescents versus adults with AN and BN, with the exception of the comparison between adolescents with BN and adults with BN [77].

Moreover, significant results have been obtained when computing the invariant global strength for many pairs of networks, among those: groups of individuals with low versus high levels of overvaluation of shape and weight, with the latter resulting in higher connectivity [43]; clinical versus nonclinical samples, with a higher density in the former [82]; groups of individuals split based on the median value of the EDE-Q global scores at admission and discharge, with denser networks at admission predicting less change in ED symptomatology during treatment [79]; pre- to post treatment networks, with increased connectivity in the latter [52]; admission against discharge network, with decreased connectivity in the latter [36]; adolescents with AN versus adolescents with BN, adolescents with AN and adults with BN, both cases with higher global strength in the AN sample [77]; men with core ED symptoms versus men without them [47]; men versus women [71]; and across developmental stages [38].

Finally, having found a significant variation in the network structures, Calugi et al. [36] also tested the change in weight of links from admission to discharge. He identified few connections that were stronger at baseline than at discharge, namely *feelings of fatness* and *desiring weight loss*, *BMI* and *vomiting to control shape or weight*, and others whose relationship grew at discharge, namely *food preoccupation* and *desiring weight loss*, *fear of losing control overeating* and *vomiting to control shape or weight*, *feelings of fatness* and *dissatisfaction with weight and shape*. Schlegl et al. [77] reported instead the percentage of edges that were significantly different in each pair of networks which resulted to have significant invariant network structure statistics. The values found ranged from 2.56% for the adolescents with AN versus adults with AN comparison to 10% for the adolescents with AN versus adults with BN comparison.

Remarkably, many studies reported no differences in network structure nor in global strength with regards to different ED diagnoses [42,50,61], age [34,35,75], and sex [75,76].

One study assessing the network differences from admission to discharge, although not finding any significant changes in the global strength, reported a significant effect of time on symptom severity, indicating decreases in ED, depression, and anxiety symptoms, with medium to large effect sizes [79]. These results were assessed through repeated measures multivariate analysis of variance (RM MANOVA), which is a statistical technique to determine the degree to which multiple dependent variables (e.g., total scores of psychometric assessment tests) vary across time points.

## Discussion

Throughout this work, we first recalled the recent and promising field of psychometric network analysis and we then outlined a comprehensive review of its methods and state-of-the-art best practices applied to the processing and study of psychometric data related to EDs. Most of the reviewed studies were based on cross-sectional data retrieved from structured psychometric questionnaires administered to subpopulations of individuals diagnosed with an ED disorder. The most widely used questionnaires to assess ED specific symptoms were EDE-Q and EDI-2, which also accounts for general psychological factors. Other questionnaires widely used to assess nonspecific ED symptoms were SCL-90 and BDI. Only in a few cases, mostly conducted by Levinson and colleagues [57–59], the analysis was carried out on panel data collected by means of EMA methods or repeated administration of one or more specific questionnaires to the same sample at different time points. These were the only studies that were able to answer research questions about the dynamic of symptom networks and the intraindividual network structure. Due to the limited number of publications and the considerable clinical implications, our conclusions suggest that future research should give this issue more focus.

With regard to the general characteristics of the participants, the most blatant peculiarity is surely the clear prevalence of female patients. Only one study [47] reported a greater percentage of male participants that, however, were not recruited in a clinical setting.

Almost all studies included in their analysis a node selection step to eliminate redundant items and obtain more accurate results. Those relying on cross-sectional data mainly used a Gaussian Graphical Model with LASSO regularization technique to estimate an undirected symptom network. Only one study [45] was found to employ non-regularized methods, such as association and concentration graphs, and only one [73] produced a directed Bayesian network. The popularity of GGM in recent psychological research is well known [166], but concerns about its generalizability and replicability by using LASSO regularization have been raised due to the relatively small size of psychological datasets [167] and to the type of variables chosen for the analysis. Therefore, a major attention from researchers should be paid to verify that data meets the assumptions of GGM and, if not, choose in favor of more suitable techniques, such as MGM in the case of variables following different distributions or non-regularized methods in the case of datasets with number of parameters much smaller than the number of observations (see, for example, [116,168]).

The parallel estimation of symptom networks on different subsamples was achieved in few cases through the FGL technique [47,62,77,78]. Finally, different studies based on panel data were also found to employ mlVAR to estimate between-subject networks and graphicalVAR to estimate temporal and contemporaneous networks [57–59].

The network description step was focused on the identification of the core symptoms and of the bridge symptoms in case of research questions concerning comorbidities. As for the first point, our survey suggest that the most used network centrality measures are strength and expected influence: the application of these indices highlighted that both specific and nonspecific ED symptoms are central for the development and maintenance of ED psychopathology, in particular *shape and weight overvaluation*, *body dissatisfaction*, *fear of weight gain*, *drive for thinness*, *ineffectiveness*, *lack of interoceptive awareness*, and *social insecurity*. Similarly, the most used network measures for the identification of bridge symptoms are bridge strength and bridge expected influence. These indices revealed that *avoidance of social eating* and *lack of self-confidence* were found to bridge ED with anxiety disorders, whereas *feelings of worthlessness*, *having a negative reaction to wanting to weigh oneself weekly* and *not wanting to eat in social situations* were found to bridge ED with depression. When exploring the relationship with the external field, *emotional abuse during childhood* has been identified as a highly influential variable for the development of any ED [65,67]. In all longitudinal studies ED specific symptoms like *overvaluation of weight and shape* and *fear of weight gain* reported the highest in-strength and out-strength centrality. Finally, the pre- to post-treatment comparison revealed that central symptoms remained constant across all time points, with more severe symptom levels associated with a lower possibility of recovery, higher clinical impairment [44,69]. A change in the role of certain symptoms was found instead by Hilbert et al. [52] and Calugi et al. [36].

Network stability analysis has been conducted in almost all papers under review (explicitly reported in 50 out of 57). In particular, almost all authors employed the bootstrap methods previously described to compute the accuracy of the estimated edge weights, the stability of the order of centrality indices, and difference between specific edge weights or centrality indices. One common finding is that strength and expected influence generally performs much better, i.e., are more stable, than closeness and betweenness centrality and can thus be assumed to be more reliable indices of the centrality of symptoms. Formally, a major difference in the computation of these measures is that strength and expected influence consider, for each node, only its adjacent nodes, whereas closeness and betweenness are computed based on paths of arbitrary length. Therefore, a possible explanation for the low stability of the latter measures is that the patterns of influence among distant symptoms and psychological traits are extremely variable in terms of population, meaning that small variations in the sample composition produce very different patterns. On the contrary, the direct effects of each symptom on any other in the network do not change significantly by taking an arbitrarily small subsample of the original observations. For a clinical perspective, the detection of symptoms and psychological variables with high closeness and betweenness centrality could be extremely meaningful, since it would help them target those factors responsible for the development of comorbidities and thus avoid a worsening in the severity of the clinical picture of the patient. Because of the inadequate results obtained from cross-sample data in this context, further research exploring the features of individual symptom networks is recommendable.

The NCT was employed to reveal differences in the symptom networks of samples with different characteristics. In particular, our study reported many cases in which similarities in network structures were found, although with different levels of connectivity. An important note should be mentioned here about the global strength of pre- and posttreatment networks. According to the network theory of psychopathology, effective treatment should lead to a decrease in network connectivity and its self-sustaining character, but studies assessing this assumption reported contrasting results that either supported it [36] or not [52], suggesting that further research on the predictive value of network variables in the therapeutic outcome is needed.

As regards the software tools available, our data highlight the fact that the state-of-the-art procedures are all based on a collection of *R* packages specifically designed for the network analysis of psychometric data. To our knowledge, no study employed other software commonly used in network science, such as Cytoscape [169], Pajek [170], or Python libraries, whose integration might bring significant contributions to the field of psychometric network analysis (see, for example, [171]). In particular, the use of free, general purpose and distinctly "user friendly" tools such as Cytoscape could bring clinicians closer to the network approach, and network experts to the field of psychopathologies, and facilitate a broader, more aware and interdisciplinary use of these potentially very effective methods.

## Conclusion

The aim of this study was to present characteristics, usage and output of the main network-based methods applied to EDs psychometric data, including their similarities and differences. We here presented the largest and most comprehensive review about psychometric network analysis methods up to date, taking into consideration 57 works published from 2016 (our queries did not retrieve any psychometric network analysis paper published before that date) to early 2022, and allowing to fill some of the gaps present and recognized in previous reviews in terms of article coverage and specific focus on network-based methodologies.

One major contribution of this article that was missing in the previous reviews is in the inclusion of studies based on panel data. This kind of data is fundamental to estimate temporal and intraindividual networks, which both might lead to significant clinical findings, such as the psychological dynamics responsible for the oscillation among different EDs during the lifetime of a patient, or the specific psychological variables that, due to the unique personal history of each patient, are involved in the maintenance and development of comorbidities and should be thus targeted for clinical intervention.

In conclusion, what emerged from our study is that there is a general agreement in the methodologies to be used for psychometric network analysis that depict a coherent image describing a strong symptom network where both specific and nonspecific ED symptoms are central for the development and maintenance of ED psychopathology. However, this image is still incomplete. Firstly, because the results of the present systematic review suggest that methodological developments are still needed to model both temporal and intraindividual symptoms and to integrate different input information into one single network structure. A possible direction to accomplish these tasks might be the multilayer network approach [172], according to which a complex system can be modeled as a network of networks, in other words, as a set of multiple layers with connections between and within them [173] In the case of mental disorders, one can think of extending the study of symptom networks with other entities (such as genetic factors, brain structure and functional connectivity, environmental factors) as layers in a multilayer network. An attempt to implement this approach has already been proposed to integrate multiple levels of personality, namely neural and psychological constituents [174], but an application to psychopathologies is also advised [175].

Furthermore, even though the studies here collected and reviewed suggest that network methods can be useful and effective in clinical practice, to the best of our knowledge the great majority of such studies have not undergone experimental verification yet.

## Supporting information

S1 Checklist PRISMA 2020 checklist for abstract. (DOCX)

S2 Checklist PRISMA 2020 checklist. (DOCX)

S1 Appendix Historical recap of the network approach to psychopathology. (DOCX)

## References

[1] Page MJ, McKenzie JE, Bossuyt PM, Boutron I, Hoffmann TC, Mulrow CD, et al. The PRISMA 2020 statement: an updated guideline for reporting systematic reviews. BMJ [Internet]. 2021;n71. Available from: doi: 10.1136/bmj.n71 33782057 PMC8005924

[2] Association AP. Diagnostic and statistical manual of mental disorders [Internet]. 5th ed. 2013. Available from: 10.1176/appi.books.9780890425596.

[3] Borsboom D, Cramer AOJ. Network analysis: an integrative approach to the structure of psychopathology. Annu Rev Clin Psychol [Internet]. 2013;9:91–121. Available from: doi: 10.1146/annurev-clinpsy-050212-185608 23537483

[4] Jones PJ, Heeren A, McNally RJ. Commentary: A network theory of mental disorders. Front Psychol [Internet]. 2017;8:1305. Available from: doi: 10.3389/fpsyg.2017.01305 28824490 PMC5539126

[5] McNally RJ. Network analysis of psychopathology: Controversies and challenges. Annu Rev Clin Psychol [Internet]. 2021;17:31–53. Available from: doi: 10.1146/annurev-clinpsy-081219-092850 33228401

[6] Borsboom D. A network theory of mental disorders. World Psychiatry [Internet]. 2017;16(1):5–13. Available from: doi: 10.1002/wps.20375 28127906 PMC5269502

[7] McNally RJ. Can network analysis transform psychopathology? Behav Res Ther [Internet]. 2016;86:95–104. Available from: doi: 10.1016/j.brat.2016.06.006 27424882

[8] Bollobás B. Modern Graph Theory. New York: Springer-Verlag; 1998.

[9] Barabási AL, Oltvai Z. Network biology: understanding the cell’s functional organization. Nat Rev Genet [Internet]. 2004;5:101–13. Available from: doi: 10.1038/nrg1272 14735121

[10] Barabasi AL, Gulbahce N, J L. Network medicine: a network-based approach to human disease. Nat Rev Genet [Internet]. 2011;12:56–68. Available from: doi: 10.1038/nrg2918 21164525 PMC3140052

[11] Barabási AL, Pósfai M. Network science [Internet]. 2016. Available from: http://barabasi.com/networksciencebook/.

[12] Silverman EK, Schmidt HHHW, Anastasiadou E, Altucci L, Angelini M, Badimon L, et al. Molecular networks in Network Medicine: Development and applications. Wiley Interdiscip Rev Syst Biol Med [Internet]. 2020 Nov;12(6):e1489. Available from: doi: 10.1002/wsbm.1489 32307915 PMC7955589

[13] Tieri P, Farina L, Petti M, Astolfi L, Paci P, Castiglione F. Network Inference and Reconstruction in Bioinformatics. In: Encyclopedia of Bioinformatics and Computational Biology [Internet]. 2019. Available from: 10.1016/B978-0-12-809633-8.20290-2.

[14] Tosi G, Borsani C, Castiglioni S, Daini R, Franceschi M, Romano D. Complexity in neuropsychological assessments of cognitive impairment: A network analysis approach. Cortex [Internet]. 2020 Mar;124:85–96. Available from: doi: 10.1016/j.cortex.2019.11.004 31846889

[15] Cramer AOJ, Waldorp LJ, Maas HLJ, Borsboom D. Comorbidity: A network perspective. Behav Brain Sci [Internet]. 2010;33(2–3):137–50. Available from: doi: 10.1017/S0140525X09991567 20584369

[16] Cramer AOJ. The glue of (ab) normal mental life: Networks of interacting thoughts, feelings and behaviors. Universiteit van Amsterdam; 2013.

[17] Borkulo C, Boschloo L, Borsboom D, Penninx BWJH, Waldorp LJ, Schoevers RA. Association of symptom network structure with the course of [corrected] depression. JAMA Psychiatry [Internet]. 2015;72(12):1219–26. Available from: doi: 10.1001/jamapsychiatry.2015.2079 26561400

[18] Borsboom D, Deserno MK, Rhemtulla M, Epskamp S, Fried EI, McNally RJ, et al. Network analysis of multivariate data in psychological science. Nature Reviews Methods Primers [Internet]. 2021;1(1):1–18. Available from: 10.1038/s43586-021-00055-w.

[19] Christensen AP, Kenett YN, Aste T. Network structure of the Wisconsin Schizotypy Scales–Short Forms: Examining psychometric network filtering approaches. Behav Res Methods [Internet]. 2018;50:2531–50. Available from: doi: 10.3758/s13428-018-1032-9 29520631

[20] Forbes MK, Wright AGC, Markon KE, Krueger RF. Evidence that psychopathology symptom networks have limited replicability. J Abnorm Psychol [Internet]. 2017;126(7):969–88. Available from: doi: 10.1037/abn0000276 29106281 PMC5749927

[21] Letina S, Blanken T, Borsboom D, Deserno M. Expanding network analysis tools in psychological networks: Minimal spanning trees, participation coefficients, and motif analysis applied to a network of 26 psychological attributes [Internet]. 2018. Available from: 10.31234/osf.io/pbg26.

[22] Borsboom D, Robinaugh DJ, Psychosystems G, Rhemtulla M, Cramer AOJ. Robustness and replicability of psychopathology networks. World Psychiatry [Internet]. 2018;17(2):143–4. Available from: doi: 10.1002/wps.20515 29856550 PMC5980315

[23] Christensen AP, Golino H. Estimating the stability of psychological dimensions via bootstrap exploratory graph analysis: A monte carlo simulation and tutorial. Psych [Internet]. 2021;3(3):479–500. Available from: 10.3390/psych3030032.

[24] Epskamp S, Borsboom D, Fried EI. Estimating psychological networks and their accuracy: A tutorial paper. Behav Res Methods [Internet]. 2018;50(1):195–212. Available from: doi: 10.3758/s13428-017-0862-1 28342071 PMC5809547

[25] Fried EI, Cramer AOJ. Moving forward: Challenges and directions for psychopathological network theory and methodology. Perspect Psychol Sci [Internet]. 2017;12(6):999–1020. Available from: doi: 10.1177/1745691617705892 28873325

[26] Fried EI, Borkulo CD, Epskamp S. On the importance of estimating parameter uncertainty in network psychometrics: A response to forbes et al. Multivariate Behav Res [Internet]. 2021;56(2):243–8. Available from: 10.1080/00273171.2020.1746903. 32264714

[27] Fried EI, Epskamp S, Nesse RM, Tuerlinckx F, Borsboom D. What are good depression symptoms? Comparing the centrality of dsm and non-dsm symptoms of depression in a network analysis. J Affect Disord [Internet]. 2016;189:314–20. Available from: https://www.sciencedirect.com/science/article/pii/S0165032715305383?casa_token=0Feqe-i9tAwAAAAA:UsTlN7pt2LfQ6d5_nRhgu2djPfTFzlac_z0n83qNyBT_Y7YMPtk_6xltBXNe3mTfmdg2YXK82W0. doi: 10.1016/j.jad.2015.09.005 26458184

[28] Levinson CA, Vanzhula IA, Brosof LC, Forbush K. Network analysis as an alternative approach to conceptualizing eating disorders: Implications for research and treatment. Curr Psychiatry Rep [Internet]. 2018;20(9):67. Available from: doi: 10.1007/s11920-018-0930-y 30079431

[29] Smith KE, Crosby RD, Wonderlich SA, Forbush KT, Mason TB, Moessner M. Network analysis: An innovative framework for understanding eating disorder psychopathology. Int J Eat Disord [Internet]. 2018;51(3):214–22. Available from: doi: 10.1002/eat.22836 29451959 PMC5946321

[30] Monteleone AM, Cascino G. A systematic review of network analysis studies in eating disorders: Is time to broaden the core psychopathology to non specific symptoms. Eur Eat Disord Rev [Internet]. 2021;29(4):531–47. Available from: doi: 10.1002/erv.2834 33942439 PMC8251923

[31] Aloi M, Rania M, Carbone EA, Caroleo M, Calabrò G, Zaffino P, et al. Metacognition and emotion regulation as treatment targets in binge eating disorder: a network analysis study. Journal of Eating Disorders [Internet]. 2021;9(1):22. Available from: doi: 10.1186/s40337-021-00376-x 33588943 PMC7885411

[32] Beauchamp MT, Allison KC, Lundgren JD. The nature of night eating syndrome: Using network analysis to understand unique symptomological relationships. Int J Eat Disord [Internet]. 2021;54(5):733–44. Available from: doi: 10.1002/eat.23497 33675062

[33] Bronstein MV, Everaert J, Kummerfeld E, Haynos AF, Vinogradov S. Biased and inflexible interpretations of ambiguous social situations: Associations with eating disorder symptoms and socioemotional functioning. Int J Eat Disord [Internet]. 2022;27:1–9. Available from: doi: 10.1002/eat.23688 35132668 PMC9392902

[34] Brown TA, Vanzhula IA, Reilly EE, Levinson CA, Berner LA, Krueger A, et al. Body mistrust bridges interoceptive awareness and eating disorder symptoms. J Abnorm Psychol [Internet]. 2020;129(5):445–56. Available from: doi: 10.1037/abn0000516 32202809 PMC8140607

[35] Calugi S, Sartirana M, Misconel A, Boglioli C, Dalle Grave R. Eating disorder psychopathology in adults and adolescents with anorexia nervosa: A network approach. Int J Eat Disord [Internet]. 2020 May;53(5):420–31. Available from: doi: 10.1002/eat.23270 32314382

[36] Calugi S, Dametti L, Chimini M, Grave AD, Grave RD. Change in eating‐disorder psychopathology network structure in patients with anorexia nervosa treated with intensive cognitive behavior therapy. Int J Eat Disord [Internet]. 2021;54(10):1800–9. Available from: doi: 10.1002/eat.23590 34331465

[37] Cascino G, Castellini G, Stanghellini G, Ricca V, Cassioli E, Ruzzi V, et al. The role of the embodiment disturbance in the anorexia nervosa psychopathology: A network analysis study. Brain Sciences [Internet]. 2019;9(10). Available from: doi: 10.3390/brainsci9100276 31619011 PMC6826416

[38] Christian C, Perko VL, Vanzhula IA, Tregarthen JP, Forbush KT, Levinson CA. Eating disorder core symptoms and symptom pathways across developmental stages: A network analysis. J Abnorm Psychol [Internet]. 2020 Feb;129(2):177–90. Available from: doi: 10.1037/abn0000477 31714097

[39] Christian C, Williams BM, Hunt RA, Wong VZ, Ernst SE, Spoor SP, et al. A network investigation of core symptoms and pathways across duration of illness using a comprehensive cognitive-behavioral model of eating-disorder symptoms. Psychol Med [Internet]. 2021;51(5):815–24. Available from: doi: 10.1017/S0033291719003817 31907093

[40] Cusack CE, Christian C, Drake JE, Levinson CA. A network analysis of eating disorder symptoms and co-occurring alcohol misuse among heterosexual and sexual minority college women. Addict Behav [Internet]. 2021;118:106867. Available from: doi: 10.1016/j.addbeh.2021.106867 33639368

[41] De Paoli T, Fuller-Tyszkiewicz M, Huang C, Krug I. A network analysis of borderline personality disorder symptoms and disordered eating. J Clin Psychol [Internet]. 2020;76(4):787–800. Available from: doi: 10.1002/jclp.22916 31953849

[42] De Vos JA, Radstaak M, Bohlmeijer ET, Westerhof GJ. The psychometric network structure of mental health in eating disorder patients. Eur Eat Disord Rev [Internet]. 2021;29(4):559–74. Available from: doi: 10.1002/erv.2832 33949742 PMC8252750

[43] DuBois RH, Rodgers RF, Franko DL, Eddy KT, Thomas JJ. A network analysis investigation of the cognitive-behavioral theory of eating disorders. Behav Res Ther [Internet]. 2017 Oct;97:213–21. Available from: doi: 10.1016/j.brat.2017.08.004 28826067

[44] Elliott H, Jones PJ, Schmidt U. Central Symptoms Predict Posttreatment Outcomes and Clinical Impairment in Anorexia Nervosa: A Network Analysis. Clin Psychol Sci [Internet]. 2020 Jan 1;8(1):139–54. Available from: 10.1177/2167702619865958.

[45] Forbush KT, Siew CSQ, Vitevitch MS. Application of network analysis to identify interactive systems of eating disorder psychopathology. Psychol Med [Internet]. 2016;46(12):2667–77. Available from: doi: 10.1017/S003329171600012X 27387196

[46] Forrest LN, Jones PJ, Ortiz SN, Smith AR. Core psychopathology in anorexia nervosa and bulimia nervosa: A network analysis. Int J Eat Disord [Internet]. 2018 Jul;51(7):668–79. Available from: doi: 10.1002/eat.22871 29693747

[47] Forrest LN, Perkins NM, Lavender JM, Smith AR. Using network analysis to identify central eating disorder symptoms among men. Int J Eat Disord [Internet]. 2019;52(8):871–84. Available from: doi: 10.1002/eat.23123 31228298

[48] Forrest LN, Sarfan LD, Ortiz SN, Brown TA, Smith AR. Bridging eating disorder symptoms and trait anxiety in patients with eating disorders: A network approach. Int J Eat Disord [Internet]. 2019;52(6):701–11. Available from: doi: 10.1002/eat.23070 30900758

[49] Giles S, Hughes EK, Fuller-Tyszkiewicz M, Treasure J, Fernandez-Aranda F, Karwautz AFK, et al. Bridging of childhood obsessive-compulsive personality disorder traits and adult eating disorder symptoms: A network analysis approach. Eur Eat Disord Rev [Internet]. 2022;30(2):110–23. Available from: doi: 10.1002/erv.2885 35064607

[50] Goldschmidt AB, Crosby RD, Cao L, Moessner M, Forbush KT, Accurso EC, et al. Network analysis of pediatric eating disorder symptoms in a treatment-seeking, transdiagnostic sample. J Abnorm Psychol [Internet]. 2018 Feb;127(2):251–64. Available from: doi: 10.1037/abn0000327 29528678 PMC5851474

[51] Hagan KE, Matheson BE, Datta N, L’Insalata AM, Onipede ZA, Gorrell S, et al. Understanding outcomes in family-based treatment for adolescent anorexia nervosa: a network approach. Psychol Med [Internet]. 2021;1–12. Available from: doi: 10.1017/S0033291721001604 33952357 PMC8820974

[52] Hilbert A, Herpertz S, Zipfel S, Tuschen-Caffier B, Friederich HC, Mayr A, et al. Psychopathological networks in cognitive-behavioral treatments for binge-eating disorder. Psychother Psychosom [Internet]. 2020;89(6):379–85. Available from: doi: 10.1159/000509458 32694245

[53] Kenny B, Orellana L, Fuller-Tyszkiewicz M, Moodie M, Brown V, Williams J. Depression and eating disorders in early adolescence: A network analysis approach. Int J Eat Disord [Internet]. 2021;54(12):2143–54. Available from: doi: 10.1002/eat.23627 34625986

[54] Kerr-Gaffney J, Halls D, Harrison A, Tchanturia K. Exploring relationships between autism spectrum disorder symptoms and eating disorder symptoms in adults with anorexia nervosa: A network approach. Front Psychiatry [Internet]. 2020;11:401. Available from: doi: 10.3389/fpsyt.2020.00401 32477185 PMC7235355

[55] Kinkel-Ram SS, Williams BM, Ortiz SN, Forrest L, Magee JC, Smith AR, et al. Testing intrusive thoughts as illness pathways between eating disorders and obsessive-compulsive disorder symptoms: A network analysis. Eat Disord [Internet]. 2021;1–23. Available from: doi: 10.1080/10640266.2021.1993705 34711137

[56] Levinson CA, Zerwas S, Calebs B, Forbush K, Kordy H, Watson H, et al. The core symptoms of bulimia nervosa, anxiety, and depression: A network analysis. J Abnorm Psychol [Internet]. 2017;126(3):340–54. Available from: doi: 10.1037/abn0000254 28277735 PMC5378619

[57] Levinson CA, Vanzhula I, Brosof LC. Longitudinal and personalized networks of eating disorder cognitions and behaviors: Targets for precision intervention a proof of concept study. Int J Eat Disord [Internet]. 2018;51(11):1233–43. Available from: doi: 10.1002/eat.22952 30291641

[58] Levinson CA, Vanzhula IA, Smith TW, Stice E. Group and longitudinal intra-individual networks of eating disorder symptoms in adolescents and young adults at-risk for an eating disorder. Behav Res Ther [Internet]. 2020;135:103731. Available from: doi: 10.1016/j.brat.2020.103731 33010651 PMC7688499

[59] Levinson CA, Hunt RA, Keshishian AC, Brown ML, Vanzhula I, Christian C, et al. Using individual networks to identify treatment targets for eating disorder treatment: A proof-of-concept study and initial data. Journal of Eating Disorders [Internet]. 2021;9(1):147. Available from: doi: 10.1186/s40337-021-00504-7 34736538 PMC8567590

[60] Liebman RE, Becker KR, Smith KE, Cao L, Keshishian AC, Crosby RD, et al. Network Analysis of Posttraumatic Stress and Eating Disorder Symptoms in a Community Sample of Adults Exposed to Childhood Abuse. J Trauma Stress [Internet]. 2021;34(3):665–74. Available from: doi: 10.1002/jts.22644 33370465

[61] Mares SHW, Burger J, Lemmens LHJM, van Elburg AA, Vroling MS. Evaluation of the cognitive behavioural theory of eating disorders: A network analysis investigation. Eat Behav [Internet]. 2022 Jan;44:101590. Available from: doi: 10.1016/j.eatbeh.2021.101590 34896868

[62] Martini M, Marzola E, Brustolin A, Abbate-Daga G. Feeling imperfect and imperfectly feeling: A network analysis on perfectionism, interoceptive sensibility, and eating symptomatology in anorexia nervosa. Eur Eat Disord Rev [Internet]. 2021;29(6):893–909. Available from: doi: 10.1002/erv.2863 34510651

[63] Meier M, Kossakowski JJ, Jones PJ, Kay B, Riemann BC, McNally RJ. Obsessive–compulsive symptoms in eating disorders: A network investigation. Int J Eat Disord [Internet]. 2020;53(3):362–71. Available from: doi: 10.1002/eat.23196 31749199

[64] Monteleone AM, Corsi E, Cascino G, Ruzzi V, Ricca V, Ashworth R, et al. The interaction between mentalizing, empathy and symptoms in people with eating disorders: A network analysis integrating experimentally induced and self-report measures. Cognit Ther Res [Internet]. 2020;44(6):1140–9. Available from: 10.1007/s10608-020-10126-z.

[65] Monteleone AM, Tzischinsky O, Cascino G, Alon S, Pellegrino F, Ruzzi V, et al. The connection between childhood maltreatment and eating disorder psychopathology: a network analysis study in people with bulimia nervosa and with binge eating disorder. Eating and Weight Disorders—Studies on Anorexia, Bulimia and Obesity [Internet]. 2022 Feb 1;27(1):253–61. Available from: doi: 10.1007/s40519-021-01169-6 33774786 PMC8860810

[66] Monteleone AM, Cardi V, Ambwani S, Cascino G, Albano G, Pellegrino F, et al. Network intervention analysis to assess the trajectory of change and treatment effects associated with the use of online guided self-help for anorexia nervosa. Early Interv Psychiatry [Internet]. 2021 Oct;15(5):1210–6. Available from: https://onlinelibrary.wiley.com/doi/abs/10.1111/eip.13064?casa_token=L4Ocqc1KwXEAAAAA:1E_RKYHZxcSSMcR7rs1tKxPRbFGEii44Wunr3m7keUFQPmDufCPkbv6JmETpWWzqQZ0Zf7d5GUzZ. 33058456 10.1111/eip.13064

[67] Monteleone AM, Cascino G, Pellegrino F, Ruzzi V, Patriciello G, Marone L, et al. The association between childhood maltreatment and eating disorder psychopathology: A mixed-model investigation. Eur Psychiatry [Internet]. 2019;61:111–8. Available from: doi: 10.1016/j.eurpsy.2019.08.002 31437672

[68] Monteleone AM, Mereu A, Cascino G, Criscuolo M, Castiglioni MC, Pellegrino F, et al. Re-conceptualization of anorexia nervosa psychopathology: A network analysis study in adolescents with short duration of the illness. Int J Eat Disord [Internet]. 2019;52(11):1263–73. Available from: doi: 10.1002/eat.23137 31313374

[69] Olatunji BO, Levinson C, Calebs B. A network analysis of eating disorder symptoms and characteristics in an inpatient sample. Psychiatry Res [Internet]. 2018;262:270–81. Available from: doi: 10.1016/j.psychres.2018.02.027 29477070

[70] Perez M, Perko V, Yu KY, Hernández JC, Ohrt TK, Stadheim J. Identifying central symptoms of eating disorders among ethnic and racial minority women. J Abnorm Psychol [Internet]. 2021;130(7):748–60. Available from: doi: 10.1037/abn0000695 34516171

[71] Perko VL, Forbush KT, Siew CSQ, Tregarthen JP. Application of network analysis to investigate sex differences in interactive systems of eating-disorder psychopathology. Int J Eat Disord [Internet]. 2019;52(12):1343–52. Available from: doi: 10.1002/eat.23170 31608479

[72] Ralph-Nearman C, Williams BM, Ortiz AML, Smith AR, Levinson CA. Pinpointing core and pathway symptoms among sleep disturbance, anxiety, worry, and eating disorder symptoms in anorexia nervosa and atypical anorexia nervosa. J Affect Disord [Internet]. 2021;294:24–32. Available from: doi: 10.1016/j.jad.2021.06.061 34256182

[73] Rodgers RF, DuBois R, Thiebaut S, Jaussent I, Maimoun L, Seneque M, et al. Structural differences in eating disorder psychopathology after history of childhood abuse: Insights from a bayesian network analysis. J Abnorm Psychol [Internet]. 2019;128(8):795–805. Available from: doi: 10.1037/abn0000470 31599631

[74] Rodgers RF, DuBois R, Frumkin MR, Robinaugh DJ. A network approach to eating disorder symptomatology: Do desire for thinness and fear of gaining weight play unique roles in the network? Body Image [Internet]. 2018;27:1–9. Available from: doi: 10.1016/j.bodyim.2018.07.004 30086480

[75] Sahlan RN, Williams BM, Forrest LN, Saunders JF, Fitzsimmons-Craft EE, Levinson CA. Disordered eating, self-esteem, and depression symptoms in Iranian adolescents and young adults: A network analysis. Int J Eat Disord [Internet]. 2021;54(2):132–47. Available from: doi: 10.1002/eat.23365 32865853 PMC8159574

[76] Sahlan RN, Keshishian AC, Christian C, Levinson CA. Eating disorder and social anxiety symptoms in Iranian preadolescents: a network analysis. In: Eating and Weight Disorders—Studies on Anorexia, Bulimia and Obesity [Internet]. 2021. Available from: doi: 10.1007/s40519-021-01329-8 34787832

[77] Schlegl S, Smith KE, Vierl L, Crosby RD, Moessner M, Neumayr C, et al. Using network analysis to compare diagnosis-specific and age-specific symptom networks in eating disorders. Int J Eat Disord [Internet]. 2021 Aug;54(8):1463–76. Available from: doi: 10.1002/eat.23523 33949717

[78] Smith AR, Forrest LN, Duffy ME, Jones PJ, Joiner TE, Pisetsky EM. Identifying bridge pathways between eating disorder symptoms and suicidal ideation across three samples. J Abnorm Psychol [Internet]. 2020;129(7):724–36. Available from: doi: 10.1037/abn0000553 32463262

[79] Smith KE, Mason TB, Crosby RD, Cao L, Leonard RC, Wetterneck CT, et al. A comparative network analysis of eating disorder psychopathology and co-occurring depression and anxiety symptoms before and after treatment. Psychol Med [Internet]. 2019;49(2):314–24. Available from: doi: 10.1017/S0033291718000867 29655386 PMC6310232

[80] Solmi M, Collantoni E, Meneguzzo P, Degortes D, Tenconi E, Favaro A. Network analysis of specific psychopathology and psychiatric symptoms in patients with eating disorders [Internet]. Vol. 51, International Journal of Eating Disorders. 2018. p. 680–92. Available from: 10.1002/eat.22884. 29846016

[81] Solmi M, Collantoni E, Meneguzzo P, Tenconi E, Favaro A. Network analysis of specific psychopathology and psychiatric symptoms in patients with anorexia nervosa. Eur Eat Disord Rev [Internet]. 2019;27(1):24–33. Available from: doi: 10.1002/erv.2633 30062717

[82] Vanzhula IA, Calebs B, Fewell L, Levinson CA. Illness pathways between eating disorder and post-traumatic stress disorder symptoms: Understanding comorbidity with network analysis. Eur Eat Disord Rev [Internet]. 2019;27(2):147–60. Available from: doi: 10.1002/erv.2634 30084217 PMC6361526

[83] Vanzhula IA, Kinkel-Ram SS, Levinson CA. Perfectionism and Difficulty Controlling Thoughts Bridge Eating Disorder and Obsessive-Compulsive Disorder Symptoms: A Network Analysis. J Affect Disord [Internet]. 2021;283:302–9. Available from: doi: 10.1016/j.jad.2021.01.083 33578342

[84] Vervaet M, Puttevils L, Hoekstra RHA, Fried E, Vanderhasselt MA. Transdiagnostic vulnerability factors in eating disorders: A network analysis. Eur Eat Disord Rev [Internet]. 2021;29(1):86–100. Available from: doi: 10.1002/erv.2805 33159404

[85] Wang SB, Jones PJ, Dreier M, Elliott H, Grilo CM. Core psychopathology of treatment-seeking patients with binge-eating disorder: A network analysis investigation. Psychol Med [Internet]. 2019;49(11):1923–8. Available from: doi: 10.1017/S0033291718002702 30232948 PMC7194445

[86] Wong VZ, Christian C, Hunt RA, Levinson CA. Network investigation of eating disorder symptoms and positive and negative affect in a clinical eating disorder sample. Int J Eat Disord [Internet]. 2021;54(7):1202–12. Available from: doi: 10.1002/eat.23511 33819357

[87] Garner DM. Eating Disorder Inventory 2: EDI 2; Professional Manual. Psychological Assessment Resources; 1991.

[88] Allison KC, Lundgren JD, O’Reardon JP, Martino NS, Sarwer DB, Wadden TA, et al. The Night Eating Questionnaire (NEQ): psychometric properties of a measure of severity of the Night Eating Syndrome. Eat Behav [Internet]. 2008 Jan;9(1):62–72. Available from: doi: 10.1016/j.eatbeh.2007.03.007 18167324

[89] Cooper Z, Fairburn C. The eating disorder examination: A semi-structured interview for the assessment of the specific psychopathology of eating disorders [Internet]. Vol. 6, International Journal of Eating Disorders. 1987. p. 1–8. Available from: 10.1002/1098-108x(198701)6:1<1::aid-eat2260060102>3.0.co;2-9.

[90] Forbush KT, Wildes JE, Pollack LO, Dunbar D, Luo J, Patterson K, et al. Development and validation of the Eating Pathology Symptoms Inventory (EPSI. Psychol Assess [Internet]. 2013;25(3):859–78. Available from: doi: 10.1037/a0032639 23815116

[91] Haynos AF, Fruzzetti AE. Initial evaluation of a single-item screener to assess problematic dietary restriction. Eat Weight Disord [Internet]. 2015 Sep;20(3):405–13. Available from: doi: 10.1007/s40519-014-0161-0 25412874 PMC5904791

[92] Fairburn CG, Beglin SJ. Assessment of eating disorders: interview or self-report questionnaire? Int J Eat Disord [Internet]. 1994;16(4):363–70. Available from: 10.1002/1098-108x(199412)16:4. 7866415

[93] Garner DM. EDI-3, eating disorder inventory-3: Professional manual. Psychological Assessment Resources, Incorporated; 2004.

[94] Anderluh MB, Tchanturia K, Rabe-Hesketh S, Treasure J. Childhood obsessive-compulsive personality traits in adult women with eating disorders: defining a broader eating disorder phenotype. Am J Psychiatry [Internet]. 2003 Feb;160(2):242–7. Available from: doi: 10.1176/appi.ajp.160.2.242 12562569

[95] Stice E, Telch CF, Rizvi SL. Development and validation of the Eating Disorder Diagnostic Scale: a brief self-report measure of anorexia, bulimia, and binge-eating disorder. Psychol Assess [Internet]. 2000 Jun;12(2):123–31. Available from: doi: 10.1037//1040-3590.12.2.123 10887758

[96] Stice E, Marti CN, Spoor S, Presnell K, Shaw H. Dissonance and healthy weight eating disorder prevention programs: long-term effects from a randomized efficacy trial. J Consult Clin Psychol [Internet]. 2008 Apr;76(2):329–40. Available from: doi: 10.1037/0022-006X.76.2.329 18377128 PMC2677629

[97] Maloney MJ, McGuire JB, Daniels SR. Reliability testing of a children’s version of the Eating Attitude Test. J Am Acad Child Adolesc Psychiatry [Internet]. 1988 Sep;27(5):541–3. Available from: doi: 10.1097/00004583-198809000-00004 3182615

[98] Garner DM, Olmstead MP, Polivy J. Development and validation of a multidimensional eating disorder inventory for anorexia nervosa and bulimia. Int J Eat Disord [Internet]. 1983;2(2):15–34. Available from: 10.1002/1098-108x(198321)2:2.

[99] Levinson CA, Brosof LC, Vanzhula I, Christian C, Jones P, Rodebaugh TL, et al. Social anxiety and eating disorder comorbidity and underlying vulnerabilities: Using network analysis to conceptualize comorbidity. Int J Eat Disord [Internet]. 2018;51(7):693–709. Available from: doi: 10.1002/eat.22890 30102777

[100] Bos EH, Wanders RBK. Group-level symptom networks in depression. JAMA Psychiatry [Internet]. 2016;73(4):411. Available from: doi: 10.1001/jamapsychiatry.2015.3103 26914967

[101] Molenaar PCM. A manifesto on psychology as idiographic science: Bringing the person back into scientific psychology, this time forever [Internet]. 2009. Available from: 10.1207/s15366359mea0204_1.

[102] Striegel-Moore RH, Rosselli F, Perrin N, DeBar L, Wilson GT, May A, et al. Gender difference in the prevalence of eating disorder symptoms. Int J Eat Disord [Internet]. 2009;42(5):471–4. Available from: doi: 10.1002/eat.20625 19107833 PMC2696560

[103] Whiteman H. Why are women more vulnerable to eating disorders. Brain study sheds light Medical News Today [Internet]. 2016; Available from: http://www.medicalnewstoday.com/articles/313466.php.

[104] Hsu LKG. The gender gap in eating disorders: Why are the eating disorders more common among women? Clin Psychol Rev [Internet]. 1989;9(3):393–407. Available from: 10.1016/0272-7358(89)90063-9.

[105] Tregarthen JP, Lock J, Darcy AM. Development of a smartphone application for eating disorder self-monitoring. Int J Eat Disord [Internet]. 2015 Nov;48(7):972–82. Available from: https://onlinelibrary.wiley.com/doi/10.1002/eat.22386. 26213130 10.1002/eat.22386

[106] Derogatis LR, Cleary PA. Confirmation of the dimensional structure of the scl-90: A study in construct validation. J Clin Psychol [Internet]. 1977;33(4):981–9. Available from: 10.1002/1097-4679(197710)33:4.

[107] Derogatis LR, Unger R. Symptom Checklist-90-Revised. In: The Corsini Encyclopedia of Psychology [Internet]. 2010. Available from: 10.1002/9780470479216.corpsy0970.

[108] Beck AT, Steer RA, Brown GK. Beck depression inventory (bdi-ii [Internet]. second. Psychological CT, Ed), editors. 1996. Available from: https://books.google.com/books/about/Beck_Depression_Inventory_BDI_II_Second.html?hl=&id=b-uHmwEACAAJ.

[109] Jones PJ. Networktools: Tools for identifying important nodes in networks. R package version. 2018;1(0):10–1155.

[110] Epskamp S, Cramer AOJ, Waldorp LJ, Schmittmann VD, Borsboom D. Qgraph: Network visualizations of relationships in psychometric data. J Stat Softw [Internet]. 2012;48(4). Available from: 10.18637/jss.v048.i04.

[111] Scutari M. Learning bayesian networks with the bnlearn r package [Internet]. 2009, 2009/8/26. Available from: http://arxiv.org/abs/0908.3817.

[112] Epskamp S, Fried EI. A tutorial on regularized partial correlation networks. Psychol Methods [Internet]. 2018;23(4):617–34. Available from: doi: 10.1037/met0000167 29595293

[113] Tibshirani R. Regression Shrinkage and Selection via the Lasso. Journal of the Royal Statistical Society Series B (Methodological [Internet]. 1996;58(1):267–88. Available from: http://www.jstor.org/stable/2346178.

[114] Chen J, Chen Z. Extended bayesian information criteria for model selection with large model spaces. Biometrika [Internet]. 2008;95(3):759–71. Available from: 10.1093/biomet/asn034.

[115] Bork R, Borkulo C, Waldorp L, Cramer A, Borsboom D. Network models for clinical psychology. Stevens’ Handbook of Experimental Psychology and Cognitive Neuroscience [Internet]. 2018;5:1–35. Available from: 10.1002/9781119170174.epcn518.

[116] Williams DR, Rast P. Back to the basics: Rethinking partial correlation network methodology. British Journal if Mathematical and Statistical Psychology [Internet]. 2020;73(2):187–212. Available from: 10.1111/bmsp.12173. PMC8572131 31206621

[117] Altenbuchinger M, Weihs A, Quackenbush J, Grabe HJ, Zacharias HU. Gaussian and Mixed Graphical Models as (multi-)omics data analysis tools. Biochim Biophys Acta Gene Regul Mech [Internet]. 2020 Jun;1863(6):194418. Available from: doi: 10.1016/j.bbagrm.2019.194418 31639475 PMC7166149

[118] Ogarrio JM, Spirtes P, Ramsey J. A hybrid causal search algorithm for latent variable models. In: Conference on probabilistic graphical models. PMLR; 2016. p. 368–79. PMC5325717 28239434

[119] Richardson T. Models of Feedback: Interpretation and Discovery. Department of Philosophy, Carnegie Mellon University; 1996.

[120] Danaher P, Wang P, Witten DM. The joint graphical lasso for inverse covariance estimation across multiple classes. J R Stat Soc Series B Stat Methodol [Internet]. 2014 Mar;76(2):373–97. Available from: doi: 10.1111/rssb.12033 24817823 PMC4012833

[121] Costantini G, Richetin J, Preti E, Casini E, Epskamp S, Perugini M. Stability and variability of personality networks. A tutorial on recent developments in network psychometrics. Pers Individ Dif [Internet]. 2019 Jan 1;136:68–78. Available from: https://www.sciencedirect.com/science/article/pii/S0191886917304002.

[122] Fried EI, Eidhof MB, Palic S, Costantini G, Huisman-van Dijk HM, Bockting CLH, et al. Replicability and generalizability of posttraumatic stress disorder (ptsd) networks: A cross-cultural multisite study of ptsd symptoms in four trauma patient samples. Clin Psychol Sci [Internet]. 2018;6(3):335–51. Available from: doi: 10.1177/2167702617745092 29881651 PMC5974702

[123] Epskamp S, Waldorp LJ, Mõttus R, Borsboom D. The gaussian graphical model in cross-sectional and time-series data. Multivariate Behav Res [Internet]. 2018;53(4):453–80. Available from: doi: 10.1080/00273171.2018.1454823 29658809

[124] Epskamp S, Borkulo CD, Veen DC, Servaas MN, Isvoranu AM, Riese H, et al. Personalized network modeling in psychopathology: The importance of contemporaneous and temporal connections. Clin Psychol Sci [Internet]. 2018;6(3):416–27. Available from: doi: 10.1177/2167702617744325 29805918 PMC5952299

[125] Krieke L, Emerencia AC, Bos EH, Rosmalen JG, Riese H, Aiello M, et al. Ecological momentary assessments and automated time series analysis to promote tailored health care: A proof-of-principle study. JMIR Res Protoc [Internet]. 2015;4(3):100. Available from: doi: 10.2196/resprot.4000 26254160 PMC4705023

[126] Granger CWJ. Investigating causal relations by econometric models and cross-spectral methods. Econometrica [Internet]. 1969;37(3):424. Available from: 10.2307/1912791.

[127] Wild B, Eichler M, Friederich HC, Hartmann M, Zipfel S, Herzog W. A graphical vector autoregressive modelling approach to the analysis of electronic diary data. BMC Med Res Methodol [Internet]. 2010;10:28. Available from: doi: 10.1186/1471-2288-10-28 20359333 PMC2869334

[128] Epskamp S, Deserno MK, Bringmann LF. Mlvar: Multi-level vector autoregression. R package version. 2017;0(4):4.

[129] Bringmann LF, Vissers N, Wichers M, Geschwind N, Kuppens P, Peeters F, et al. A network approach to psychopathology: New insights into clinical longitudinal data. PLoS One [Internet]. 2013;8(4):60188. Available from: doi: 10.1371/journal.pone.0060188 23593171 PMC3617177

[130] Luiselli JK, Fischer AJ. Computer-Assisted and Web-Based Innovations in Psychology, Special Education, and Health [Internet]. Academic Press; 2016. 408 p. Available from: https://play.google.com/store/books/details?id=NwLSBgAAQBAJ.

[131] Stone AA, Shiffman S. Ecological momentary assessment (EMA) in behavorial medicine. Ann Behav Med [Internet]. 1994;16(3):199–202. Available from: https://psycnet.apa.org/fulltext/1995-10701-001.pdf.

[132] Opsahl T, Agneessens F, Skvoretz J. Node centrality in weighted networks: Generalizing degree and shortest paths. Soc Networks [Internet]. 2010;32(3):245–51. Available from: 10.1016/j.socnet.2010.03.006.

[133] Freeman LC. Centrality in social networks conceptual clarification. Soc Networks [Internet]. 1978 Jan;1(3):215–39. Available from: https://linkinghub.elsevier.com/retrieve/pii/0378873378900217.

[134] Robinaugh DJ, Millner AJ, McNally RJ. Identifying highly influential nodes in the complicated grief network. J Abnorm Psychol [Internet]. 2016;125(6):747–57. Available from: doi: 10.1037/abn0000181 27505622 PMC5060093

[135] Bringmann LF, Elmer T, Epskamp S, Krause RW, Schoch D, Wichers M, et al. What do centrality measures measure in psychological networks? J Abnorm Psychol [Internet]. 2019 Nov;128(8):892–903. Available from: doi: 10.1037/abn0000446 31318245

[136] Teicher MH, Samson JA. Childhood maltreatment and psychopathology: A case for ecophenotypic variants as clinically and neurobiologically distinct subtypes. Am J Psychiatry [Internet]. 2013;170(10):1114–33. Available from: doi: 10.1176/appi.ajp.2013.12070957 23982148 PMC3928064

[137] Monteleone AM, Cascino G, Ruzzi V, Pellegrino F, Patriciello G, Barone E, et al. Emotional traumatic experiences significantly contribute to identify a maltreated ecophenotype sub-group in eating disorders: Experimental evidence. Eur Eat Disord Rev [Internet]. 2021;29(2):269–80. Available from: doi: 10.1002/erv.2818 33378110

[138] Jones PJ, Ma R, McNally RJ. Bridge centrality: A network approach to understanding comorbidity. Multivariate Behav Res [Internet]. 2021;56(2):353–67. Available from: doi: 10.1080/00273171.2019.1614898 31179765

[139] Mehling WE, Price C, Daubenmier JJ, Acree M, Bartmess E, Stewart A. The multidimensional assessment of interoceptive awareness (maia. PLoS One [Internet]. 2012;7(11):48230. Available from: doi: 10.1371/journal.pone.0048230 23133619 PMC3486814

[140] Lovibond SH, Lovibond PF. Manual for the depression anxiety stress scales. Psychology Foundation of Australia; 1996.

[141] Henry JD, Crawford JR. The short-form version of the depression anxiety stress scales (dass-21): Construct validity and normative data in a large non-clinical sample. British Journal of Clinical Psychology [Internet]. 2005;44(Pt 2):227–39. Available from: 10.1348/014466505X29657. 16004657

[142] Dziobek I, Fleck S, Kalbe E, Rogers K, Hassenstab J, Brand M, et al. Introducing masc: A movie for the assessment of social cognition. J Autism Dev Disord [Internet]. 2006;36(5):623–36. Available from: doi: 10.1007/s10803-006-0107-0 16755332

[143] Coll MP, Viding E, Rütgen M, Silani G, Lamm C, Catmur C, et al. Are we really measuring empathy? Proposal for a new measurement framework. Neurosci Biobehav Rev [Internet]. 2017;83:132–9. Available from: doi: 10.1016/j.neubiorev.2017.10.009 29032087

[144] Mackes NK, Golm D, O’Daly OG, Sarkar S, Sonuga-Barke EJS, Fairchild G, et al. Tracking emotions in the brain–revisiting the empathic accuracy task. Neuroimage [Internet]. 2018;178:677–86. Available from: doi: 10.1016/j.neuroimage.2018.05.080 29890323 PMC6057276

[145] Frost RO, Marten P, Lahart C, Rosenblate R. The dimensions of perfectionism. Cognit Ther Res [Internet]. 1990;14(5):449–68. Available from: 10.1007/bf01172967.

[146] Kroenke K, Spitzer RL, Williams JB. The PHQ-9: Validity of a brief depression severity measure. J Gen Intern Med [Internet]. 2001;16(9):606–13. Available from: doi: 10.1046/j.1525-1497.2001.016009606.x 11556941 PMC1495268

[147] Fergus TA, Valentiner DP, McGrath PB, Gier-Lonsway SL, Kim HS. Short forms of the social interaction anxiety scale and the social phobia scale. J Pers Assess [Internet]. 2012;94(3):310–20. Available from: doi: 10.1080/00223891.2012.660291 22369684

[148] First MB, Williams JBW, Karg RS, Spitzer RL. Structured Clinical Interview for DSM-5, Research Version. Arlington, VA: American Psychiatric Association; 2015.

[149] Watson D, Clark LA, Carey G. Positive and negative affectiv-ity and their relation to anxiety and depressive disorders. J Abnorm Psychol [Internet]. 1988;97(3):346–53. Available from: doi: 10.1037//0021-843x.97.3.346 3192830

[150] Pedone R, Semerari A, Riccardi I, Procacci M, Nicolò G, Carcione A. Development of a self-report measure of metacognition: The Metacognition Self-Assessment Scale (MSAS). Instrument description and factor structure. Clinical Neuropsychiatry: Journal of Treatment Evaluation. 2017;14(3):185–94.

[151] Giromini L, Velotti P, Campora G, Bonalume L, Cesare Zavattini G. Cultural Adaptation of the Difficulties in Emotion Regulation Scale: Reliability and Validity of an Italian Version. J Clin Psychol [Internet]. 2012;68(9):989–1007. Available from: doi: 10.1002/jclp.21876 22653763

[152] Achenbach TM, Rescorla LA. Manual for the ASEBA School-age forms & profiles. Burlington, VT: University of Vermont/University of Vermont, Research Center for Children, Youth, & Families; 2007.

[153] Cloninger CR, Przybeck TR, Svrakic DM. The tridimensional personality questionnaire: U.S. Normative data Psychological Reports [Internet]. 1991;69(3):1047–57. Available from: 10.2466/pr0.1991.69.3.1047. 1784653

[154] De Jong K, Nugter MA, Lambert MJ, Burlingame GM. Handleiding voor afname en scoring van de Outcome Questionnaire OQ-45.2. Salt Lake City, UT, OQ Measures LLC; 2009.

[155] Lamers SMA, Westerhof GJ, Bohlmeijer ET, Ten Klooster PM, Keyes CLM. Evaluating the psychometric properties of the mental health Continuum-Short Form (MHC-SF. J Clin Psychol [Internet]. 2011;67(1):99–110. Available from: doi: 10.1002/jclp.20741 20973032

[156] Stanghellini G, Castellini G, Brogna P, Faravelli C, Ricca V. Identity and eating disorders (idea): A questionnaire evaluating identity and embodiment in eating disorder patients. Psychopathology [Internet]. 2012;45(3):147–58. Available from: doi: 10.1159/000330258 22398386

[157] Bernstein DP, Stein JA, Newcomb MD, Walker E, Pogge D, Ahluvalia T, et al. Development and validation of a brief screening version of the childhood trauma questionnaire. Child Abuse Negl [Internet]. 2003;27(2):169–90. Available from: doi: 10.1016/s0145-2134(02)00541-0 12615092

[158] Weathers FW, Litz BT, Keane TM, Palmieri PA, Marx BP, Schnurr PP. 2013. Available from: https://www.ptsd.va.gov/professional/assessment/adult-sr/ptsd-checklist.asp.

[159] Young JE, Klosko JS, Weishaar ME. Schema therapy: A practitioner’s guide. New York, NY: The Guilford Press; 2003.

[160] Shapurian R, Hojat M, Nayerahmadi H. Psychometric Characteristics and Dimensionality of a Persian Version of Rosenberg Self-Esteem Scale. Percept Mot Skills [Internet]. 1987;65(1):27–34. Available from: doi: 10.2466/pms.1987.65.1.27 3684462

[161] Haslbeck JMB, Waldorp LJ. How well do network models predict observations? On the importance of predictability in network models. Behav Res Methods [Internet]. 2018 Apr;50(2):853–61. Available from: doi: 10.3758/s13428-017-0910-x 28718088 PMC5880858

[162] Friedman N, Goldszmidt M, Wyner AJ. Data analysis with bayesian networks: A bootstrap approach UAI ‘99. In: Proceedings of the Fifteenth Conference on Uncertainty in Artificial Intelligence [Internet]. Stockholm, Sweden; 1999. Available from: https://dslpitt.org/uai/displayArticleDetails.jsp?mmnu=1&smnu=2&article_id=169&proceeding_id=15.

[163] Friedman N, Goldszmidt M, Wyner A. Data analysis with bayesian networks: A bootstrap approach [Internet]. 2013. Available from: http://arxiv.org/abs/1301.6695.

[164] Scutari M, Nagarajan R. Identifying significant edges in graphical models of molecular networks. Artif Intell Med [Internet]. 2013;57(3):207–17. Available from: doi: 10.1016/j.artmed.2012.12.006 23395009 PMC4070079

[165] Van Borkulo CD, Boschloo L, Kossakowski J, Tio P, Schoevers RA, Borsboom D, et al. Comparing network structures on three aspects: A permutation test. Manuscript submitted for publication [Internet]. 2017;10. Available from: https://www.researchgate.net/profile/Claudia-Van-Borkulo-2/publication/314750838_Comparing_network_structures_on_three_aspects_A_permutation_test/links/5ff5ac0345851553a022c099/Comparing-network-structures-on-three-aspects-A-permutation-test.pdf. 10.1037/met0000476 35404628

[166] Fried EI, van Borkulo CD, Cramer AOJ, Boschloo L, Schoevers RA, Borsboom D. Mental disorders as networks of problems: a review of recent insights. Soc Psychiatry Psychiatr Epidemiol [Internet]. 2017 Jan;52(1):1–10. Available from: doi: 10.1007/s00127-016-1319-z 27921134 PMC5226976

[167] Epskamp S, Isvoranu AM, Cheung MWL. Meta-analytic Gaussian Network Aggregation. Psychometrika [Internet]. 2022 Mar;87(1):12–46. Available from: doi: 10.1007/s11336-021-09764-3 34264449 PMC9021114

[168] Friedman J, Hastie T, Tibshirani R. Sparse inverse covariance estimation with the graphical lasso. Biostatistics [Internet]. 2008 Jul;9(3):432–41. Available from: doi: 10.1093/biostatistics/kxm045 18079126 PMC3019769

[169] Shannon P, Markiel A, Ozier O, Baliga NS, Wang JT, Ramage D, et al. Cytoscape: a software environment for integrated models of biomolecular interaction networks. Genome [Internet]. 2003;(,13(11)):2498–504. Available from: doi: 10.1101/gr.1239303 14597658 PMC403769

[170] Batagelj V, Mrvar A. Pajek—Program for Large Network Analysis. Connect [Internet]. 1998;21(2):47–57. Available from: http://vlado.fmf.uni-lj.si/pub/networks/doc/pajek.pdf.

[171] Costantini G, Epskamp S, Borsboom D, Perugini M, Mõttus R, Waldorp LJ, et al. State of the art personality research: A tutorial on network analysis of personality data in r. J Res Pers [Internet]. 2015;54:13–29. Available from: 10.1016/j.jrp.2014.07.003.

[172] Kivelä M, Arenas A, Barthelemy M, Gleeson JP, Moreno Y, Porter MA. Multilayer networks. Journal of Complex Networks [Internet]. 2014;2(3):203–71. Available from: 10.1093/comnet/cnu016.

[173] De Boer NS, Bruin LC, Geurts JJG, Glas G. The Network Theory of Psychiatric Disorders: A Critical Assessment of the Inclusion of Environmental Factors. Front Psychol [Internet]. 2021;12:623970. Available from: doi: 10.3389/fpsyg.2021.623970 33613399 PMC7890010

[174] Brooks D, Hulst HE, Bruin L, Glas G, Geurts JJG, Douw L. The multilayer network approach in the study of personality neuroscience. Brain Sciences [Internet]. 2020;10(12):915. Available from: 10.3390/brainsci10120915. PMC7761383 33260895

[175] Braun U, Schaefer A, Betzel RF, Tost H, Meyer-Lindenberg A, Bassett DS. From Maps to Multi-dimensional Network Mechanisms of Mental Disorders. Neuron [Internet]. 2018;97(1):14–31. Available from: doi: 10.1016/j.neuron.2017.11.007 29301099 PMC5757246

