# Supplementary material for: Network-based methods for psychometric data of eating disorders: A systematic review
Source: PLoS One. 2022 Oct 31;17(10):e0276341. doi: 10.1371/journal.pone.0276341 (PMC9621460; doi:10.1371/journal.pone.0276341)
Supplement: S1 Appendix — (DOCX) [file pone.0276341.s003.docx]

**Supplementary Materials**

**Network-Based Methods for Psychometric Data of Eating Disorders: A Systematic Review**

Clara Punzi, Manuela Petti and Paolo Tieri

1. **Appendix A: Historical Recap of the Network Approach to Psychopathology**

The network approach to psychopathology has been only recently applied to the investigation of eating disorders. However, the theoretical setting of this new perspective already started to be discussed a couple of decades ago.

In the early 2000s, Nancy S. Kim and Woo-Kyoung Ahn already showed that clinical psychologists are cognitively driven to interpret symptom patterns in terms of causal networks (137). A few years later, inspired by the philosopher Richard Boyd, the psychiatrist Kenneth Kendler suggested abandoning the traditional disease model in favor of a new quest for complex and multi-level causal mechanisms that produce, underlie, and sustain psychiatric syndromes (138,139). While answering the question of what kinds of things are psychiatric disorders, he argued that they are most likely to be “mechanistic property clusters” (MPC) kinds, which is to say, rather than having a deterministic essence, they can be described by mutually reinforcing networks of causal mechanisms (139).

In the same period, another critique to a diagnostic system based on the latent factor model was moved by Borsboom (140). He argued that one important consequence of this model is that one should assume that the property of local independence holds, which is to say, the covariance among symptoms should vanish upon conditionalizing on the presence of the disorder. Nevertheless, as many studies suggest, not only most psychiatric disorders do not satisfy the property of local independence, but also the Diagnostic and Statistical Manual of Mental Disorders (DSM) criteria often specify direct functional relations between signs and symptoms (3). Hence, the author put forward the hypothesis of an alternative view of mental disorders as causal systems (140).

Later, in order to give a more plausible explanation to comorbidity in the field of psychopathology compared to the latent factor theory, the Psychological Methods program group of the of the Psychology Research Institute of the University of Amsterdam started to develop a network approach to mental disorders and comorbidity in which symptoms are viewed as components in a network and comorbidity is hypothesized to arise from direct relations between symptoms of multiple disorders (1,4,12,14,141).

From that point on, more and more studies have been conducted by the same research group and others not only to lick the theory behind the network approach to psychopathology into shape, but also to refine and widen the required methodologies (17,20,108,133,142) and to explore its applications to some specific mental disorders (3,4).
